# Multiomics strategies for decoding seed dormancy breakdown in *Paris polyphylla*

**DOI:** 10.1186/s12870-023-04262-3

**Published:** 2023-05-11

**Authors:** Guowei Zheng, Wenchun Li, Shunzhen Zhang, Qi Mi, Wenxiu Luo, Yanli Zhao, Xiangshi Qin, Weijiao Li, Shibiao Pu, Furong Xu

**Affiliations:** 1 College of Chinese Materia Medica, Yunnan University of Chinese Medicine, Kunming, 650500 China; 2 College of Ethnic Medicines, Yunnan University of Chinese Medicine, Kunming, 650500 China; 3 Germplasm Bank of Wild Species, Kunming Institute of Botany, Chinese Academy of Sciences, Kunming, 650201 China

**Keywords:** *Paris polyphylla*, Seed dormancy, Metabolomics, Transcriptomics, Gibberellin A_3_, Gibberellin A_4_

## Abstract

**Background:**

The disruption of seed dormancy is a complicated process and is controlled by various factors. Among these factors, membrane lipids and plant hormones are two of the most important ones. *Paris polyphylla* is an important Chinese herbaceous species, and the dormancy trait of its seed limits the cultivation of this herb.

**Results:**

In this study, we investigate the global metabolic and transcriptomic profiles of *Paris polyphylla* during seed dormancy breaking. Widely targeted metabolomics revealed that lysophospholipids (lysoPLs) increased during *P. polyphylla* seed dormancy breaking. The expression of *phospholipase A2* (*PLA2*), genes correlated to the production of lysoPLs, up-regulated significantly during this process. Abscisic acid (ABA) decreased dramatically during seed dormancy breaking of *P. polyphylla*. Changes of different GAs varied during *P. polyphylla* seeds dormancy breaking, 13-OH GAs, such as GA_53_ were not detected, and GA_3_ decreased significantly, whereas 13-H GAs, such as GA_15_, GA_24_ and GA_4_ increased. The expression of *CYP707As* was not synchronous with the change of ABA content, and the expression of most *UGTs*, *GA20ox* and *GA3ox* up-regulated during seed dormancy breaking.

**Conclusions:**

These results suggest that PLA2 mediated production of lysoPLs may correlate to the seed dormancy breaking of *P. polyphylla*. The conversion of ABA to ABA-GE catalysed by UGTs may be the main cause of ABA degradation. Through inhibition the expression of genes related to the synthesis of 13-OH GAs and up-regulation genes related to the synthesis of 13-H GAs, *P. polyphylla* synthesized more bioactive 13-H GA (GA_4_) to break its seed dormancy.

**Supplementary Information:**

The online version contains supplementary material available at 10.1186/s12870-023-04262-3.

## Backgrounds

Seed dormancy is an important adaptive trait that helps plants respond to unflavoured environments. However, the seeds of most medicinal plants undergo seed dormancy, which slows plant growth. *Paris polyphylla* var. *yunnanensis* is an important traditional Chinese medicinal herbaceous species in the family Melanthiaceae [1]. The demand for and industrial production of *P. polyphylla* is increasing due to the important medicinal qualities of this species. However, wild resources have been endangered in China because of overexploitation. Therefore, artificial cultivation in the field is an effective way to meet this demand. Seeds of *P. polyphylla* are morphophysiological dormant (MPD) and cannot germinate in a short period. The breaking of seed dormancy in *P. polyphylla* was the key factor that influenced its domestication and cultivation.

Gene expression profiles during *P. polyphylla* seed dormancy breaking are well studied. During this process, differentially expressed genes (DEGs) are enriched in the pathways of carbohydrate metabolism, hormone metabolism, lipid metabolism, and hormone signalling [2–4], which indicates that carbohydrate, lipid and hormone metabolism are important for the germination of *P. polyphylla* seeds. Among them, genes related to GA synthesis (*GA20ox2*, *GA20ox3*) and ABA catabolism (CYP707A) were upregulated, whereas dormancy-related genes (*NCED*, *PP2C*) were downregulated. However, all these studies were focused on investigating the candidate genes and their correlation to the seed dormancy breaking of *P. polyphylla*, evidence of the involvement of these genes in the metabolite changes is still lacking. Therefore, the relationships between gene expression and metabolism during *P. polyphylla* seed germination still need further investigation.

Hormones are the most important metabolites that regulate seed dormancy. It is considered that abscisic acid (ABA) and gibberellin (GA) are the main regulators of seed dormancy, the balance of abscisic acid (ABA) and gibberellin (GA) regulates seed dormancy [5], and a decrease in ABA and an increase in GA were also found in germinated *P. polyphylla* seeds [6]. Through influence ABA and GA, other hormones can affect seed dormancy. Cytokinin can block ABA-induced expression of *ABI5* and promotes seed germination [7], while auxin can maintain the expression of *ABI3* and promotes seed dormancy [8]. Ethylene can down-regulate ABA accumulation and signal, and up-regulate GA accumulation and signal, which finally break seed dormancy [9]. ABA conjugation and catalytic hydroxylation are two pathways of ABA catabolism [10], and both pathways are involved in the decrease in ABA content during seed germination [11, 12]. However, little is known about ABA catabolism during *P. polyphylla* seed germination. GA_1_, GA_3_, GA_4_, and GA_7_ are bioactive GA molecules in plants [13]. The bioactivity of GA_4_ is stronger than that of GA_1_ in both Arabidopsis and rice [14–17], and GA_4_ may regulate dormancy breaking in *Syngonanthus verticillatus* seeds [18]. GA_3_ treatment is usually used as an effective way to break seed dormancy [19, 20]. However, the effect of GA_3_ on *P. polyphylla* seed germination is complex. In some studies, GA_3_ was shown to promote the seed dormancy breaking of *P. polyphylla* seeds and rhizomes [1, 21, 22], while in other studies, GA_3_ showed no effects on the seed germination of *P. polyphylla* [23, 24]. However, the metabolism of different GA molecules and their roles in the dormancy breaking of *P. polyphylla* seeds are not fully understood.

Transcriptomics and metabolomics are efficient techniques that can be used to study the gene regulation and metabolic profiles of plants at certain developmental stages [25, 26], and these two techniques were used to clarify the probable metabolic regulation process during the seed germination of *Punica granatum* [27]. In this study, the seeds of *P. polyphylla* were collected and germinated under warm temperatures. Seed at four different germination stages, air-dried seeds, seeds that germinated, seeds whose radicles had emerged, and seeds whose roots were approximately 2 cm, were defined. To identify the key metabolites and their metabolic process during dormancy breaking of *P. polyphylla* seeds, the mechanism of seed dormancy was studied through transcriptomics and widely targeted metabolomics techniques. The DEGs related to *P. polyphylla* seed dormancy disruption were confirmed via qRT‒PCR. The present study was designed to clarify the metabolic profiles of ABA and different bioactive GAs and their roles in *P. polyphylla* seed dormancy.

## Materials and methods

### Plant materials and treatments

*Paris polyphylla* var. *yunnanensis* plants were grown in Shizong (24.82822 N, 103.99084 E), Yunnan Province, China. Their seeds were harvested on 18 October 2021. After washing under tap water, the seeds were air dried for 2 days. The dry seeds were incubated in wet sand and allowed to germinate at 18/22°C (day/night) in a growth chamber. Seeds at different germination stages were harvested and immediately placed into liquid nitrogen. Three biological replicates were included in the subsequent analysis.

### Widely targeted metabolomics analysis

The freeze-dried seed samples were ground to a powder, and 100 mg of the powder was extracted with 0.6 mL of 70% aqueous methanol (4 °C) overnight. After centrifugation at 10,000 × g for 10 min, the supernatant was filtered with a microporous membrane (0.22 μm pore size) and analysed using a UPLC-ESI-MS/MS system. The UPLC conditions and materials were as follows: column, Agilent SB-C18 column (1.8 μm, 4.6 × 100 mm); solvent system, water (0.1% formic acid):acetonitrile; gradient programme, 95:5 (v/v) at 0 min, 5:95 (v/v) at 9.0 min, 5:95 (v/v) at 10 min, 95:5 (v/v) at 11.1 min, and 95:5 (v/v) at 14 min; flow rate, 0.35 mL/min; temperature, 40 °C; and injection volume, 4 µL. The effluent was alternatively connected to an ESI-triple quadrupole-linear ion trap (Q TRAP)-MS. An API 4500 Q Trap LC/MS/MS system equipped with an ESI turbo ion spray interface was used to perform linear ion trap (LIT) and triple quadrupole (QQQ) scans as previously described[28].

Identified metabolites were annotated using KEGG Compound database (http://www.kegg.jp/kegg/compound/), and then were mapped to KEGG Pathway database (http:/www.kegg.jp/kegg/pathway.htm). Pathways with significantly regulated metabolites mapped to were then fed into MSEA (metabolite sets enrichment analysis), their significance was determined by hypergeometric test’s p-values.

### Phytohormone analysis

For the analysis of phytohormones, seeds were immediately placed into liquid nitrogen and kept at -80 °C. The samples were ground to a powder, and 50 mg of powder was extracted by methanol:H_2_O:formic acid (15:4:1, v:v:v). The extraction buffer was condensed and redissolved in 100 µL of 80% methanol and then filtered (PTFE, 0.22 μm) before LC-MS/MS analysis. The LC-ESI-MS/MS conditions were as follows: HPLC column, Waters ACQUITY UPLC HSS T3 C18 (1.8 μm, 2.1 mm × 100 mm); solvent system, water (0.05% acetic acid):acetonitrile (0.05% acetic acid); gradient programme, 95:5 (v/v) at 0 min, 95:5 (v/v) at 1 min, 5:95 (v/v) at 8 min, 5:95 (v/v) at 9 min, 95:5 (v/v) at 9.1 min, 95:5 (v/v) at 12 min; flow rate, 0.35 mL/min; temperature, 40 °C; and injection volume, 2 µL. The effluent was alternatively connected to an ESI-triple quadrupole-linear ion trap (QTRAP)-MS instrument.

An AB 6500 QTRAP LC/MS/MS system equipped with an ESI turbo ion-spray interface operating in both positive and negative ion modes and controlled by Analyst 1.6 software (AB SCIEX) was used. The ESI source operation parameters were as follows: ion source, turbo spray; source temperature, 500 °C; ion spray voltage (IS), 4500 V; curtain gas (CUR), 35.0 psi; and collision gas (CAD), medium. DP and CE for individual multiple reaction monitoring (MRM) transitions were performed with further DP and CE optimization. A specific set of MRM transitions was monitored for each period according to the plant hormones eluted within the period.

The phytohormone contents were measured by MetWare (http://www.metware.cn/) based on the AB SCIEX TRAP 6500 LC‒MS/MS platform. Briefly, three replicates of each assay were assessed. Seven classes of phytohormones, including 26 kinds, were detected (Table S1, Table S2).

### Transcriptome sequencing and quantitative real-time PCR (qRT‒PCR)

RNA extraction and quality analysis, library construction, sequencing and bioinformatics analysis were performed by staff at Wuhan Metaville Biotechnology Co., Ltd. (www.metavare.cn; Wuhan, China), as previously described [29]. The expression of 21 selected DEGs related to GA metabolism, ABA metabolism and seed dormancy was determined via qRT‒qPCR. The primers used were designed based on sequence data of PacBio sequencing. Gene encoding αtubulin (*TUA*) was used as an internal reference gene, and the genes and primers used are shown in Table S3. cDNA synthesis was performed with MonScript RTIII All-in-One Mix with dsDNase (Monad, MR05101) according to the manufacturer’s instructions. qRT‒PCR was performed with a QuantiNova SYBR Green PCR Kit (QIAGEN, 208,054). The PCR initial heat activation was 95 °C for 2 min. The thermal cycling conditions were as follows: 40 cycles at 95 °C for 5 s for denaturation and 60 °C for 30 s for combined annealing and extension. All of the reactions were performed in triplicate. The relative expression levels of the DEGs were calculated using the comparative cycle threshold (Ct) method with normalization to the expression level of internal reference gene.

### Data analysis

Unsupervised Principal Component Analysis was performed by statistics function prcomp within R (www.r-project.org). The Hierarchical Cluster Analysis results of samples and metabolites was carried out by R package ComplexHeatmap. Significantly regulated metabolites between groups were determined by VIP (VIP ≥ 1) and absolute Log_2_FC (|Log2FC| ≥ 1.0). VIP values were extracted from OPL S-DA result, which also contain score plots and permutation plots, was generated using R package MetaboAnalystR. One-way ANOVA analysis was performed using SPSS 13.0. Significance was calculated using Fisher’s least significant difference (LSD).

## Results

### Four germination stages of P. polyphylla seeds

**Fig. 1:**
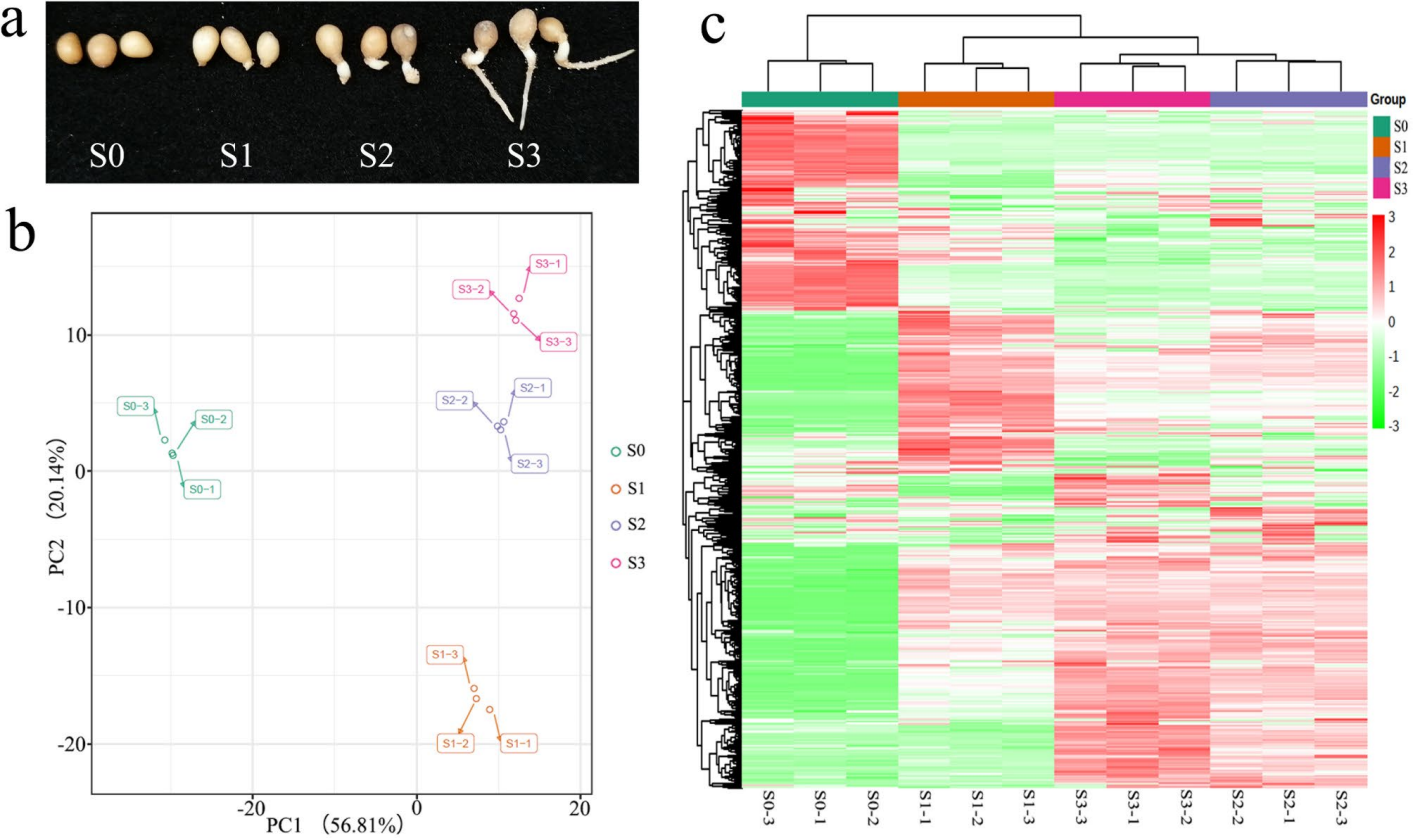
Phenotypes and SCMs during the different germination stages of *P. polyphylla*. (**a**) Phenotypes of *Paris polyphylla* seeds at different germination stages. (**b**) PCA score plot of metabolite profiles from S0, S1, S2, and S3. (**c**) Accumulation patterns of significantly changed metabolites from S0, S1, S2, and S3

The germination of *P. polyphylla* seeds was divided into four different stages (Fig. 1a). The air-dried seeds were ungerminated seeds and denoted as S0. In this stage, the embryo was not yet fully developed, and it was just an oval, primitive embryo. After stratification with warm temperature, the oval embryo fully developed; the seeds at this stage were designated as S1, and the seeds germinated at this stage. The seeds whose radicle had emerged were denoted as S2, and germinated seeds of which the roots were approximately 2 cm were denoted as S3.

### Significant changed metabolites detected during the germination of ***Paris polyphylla*** seeds

To explore the changes in metabolite levels during seed dormancy break in *P. polyphylla*, a widely targeted metabolomics method based on ultra-performance liquid chromatography and tandem mass spectrometry was used. The metabolites were assigned to 13 classes: lipids, amino acids and derivatives, phenolic acids, organic acids, nucleotides and derivatives, alkaloids, flavonoids, steroids, lignans and coumarins, terpenoids, tannins, quinones, and others. A total of 585 metabolites were identified, and the main metabolites identified were amino acids and derivatives, organic acids, lipids, nucleotides and derivatives, and phenolic acids, of which there were 86, 62, 107, 42, and 83 metabolites detected, respectively. The content of these five classes in the dry seeds was more than 70% (Table S4). The levels of most metabolite classes, such as organic acids, nucleotides and derivatives, phenolic acids, steroids, terpenoids, flavonoids, lignans and coumarins, and tannins, decreased during the germination of *P. polyphylla* seeds. The levels of amino acids and derivatives and alkaloids showed a minor increase during seed germination, while the level of lipids increased dramatically during seed germination (Table S4).

The changes in the metabolome among the seeds at different germination stages were assessed using principal component analysis (PCA). The first two principal components explained 56.81% (PC1) and 20.14% (PC2) of the overall variance of all the samples (Fig. 1b). The PCA separated seeds of different germination stages efficiently. PC1 separated the S0 stage from the other three stages, whereas PC2 separated the S1 stage from the other three stages. The samples with three replicates clustered into each group and significantly varied among different groups during the different germination stages. These results indicated that the changes in most metabolites occurred during the initiation of germination in the S1 stage and that there was significant variation in the metabolism of these samples. Hierarchical cluster analysis of the metabolites showed that the most changed metabolites occurred in the S1 stage, and the metabolite profiles of S2 and S3 were similar, which indicates that no significant changes occurred from the seed germination stage of S2 to S3 (Fig. 1c).

Metabolites for which there was a fold change ≥ 2 or ≤ 0.5 between two samples were considered significantly changing metabolites (SCMs). A total of 262 SCMs (190 increased, 72 decreased) were detected between S0 and S1 seeds, 56 SCMs (34 increased, 22 decreased) were identified between S1 and S2 seeds, and only 19 SCMs (6 increased, 13 decreased) were detected between S2 and S3 seeds (Fig. S1). Among these SCMs, 8 metabolites, namely, ethylmalonic acid, 1-methylpiperidine-2-carboxylic acid, 3-hydroxyphenylacetic acid methyl ester, 3-(3-hydroxyphenyl)-propionate acid, thymidine, 6-hydroxy-5,7,4’-trimethoxyflavone, diosgenin, and LysoPC 12:0, were differentially expressed across all the germination stages (Fig. S2, Table S5). A total of 222 SCMs were specifically detected in S0/S1, 21 SCMs were specifically detected in S1/S2, and only 2 SCMs were specifically detected in S2/S3 (Fig. S2).

**Fig. 2:**
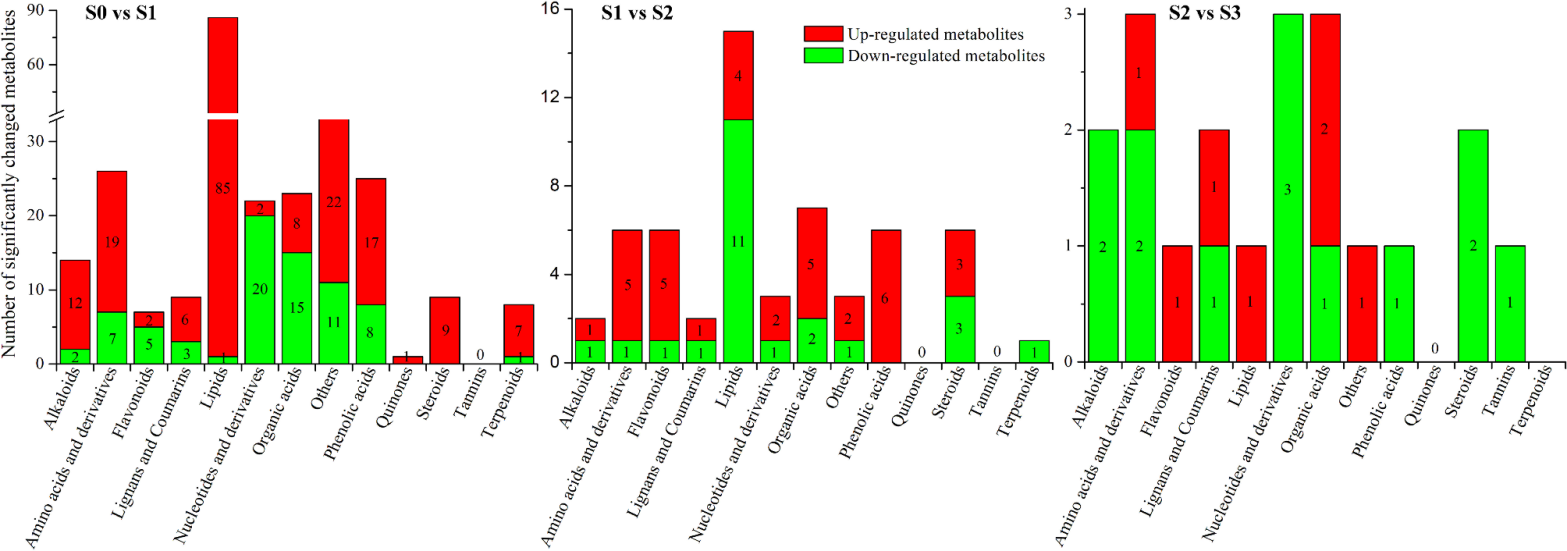
Number of SCMs during the different germination stages of *P. polyphylla*

During the primary germination stage (from S0 to S1), the most changed SCMs were lipids, of which 85 lipids increased in abundance in this process, whereas only 1 lipid (13 S-hydroxy-9Z,11E,15Z-octadecatrienoic acid) decreased in abundance (Fig. 2, Table S5). The decreased lipids were free fatty acids, which may be precursors of other lipids. Four coumarins, 6,7-dimethoxy-4-methylcoumarin, o-feruloyl 4-hydroxycoumarin, 1-methoxyphaseollin, and 4-hydroxycoumarin di-glucoside, increased during this stage, and 1 coumarin, namely, 5,7-dimethoxycoumarin, decreased (Table S5). In the stage of radicle emergence (from S1 to S2), lipids were also the most significantly changed metabolites; however, 11 lipids decreased in abundance in this process, and 4 increased, which was lower than that at the primary germination stage. One coumarin, 1-methoxyphaseollin, decreased in this process (Fig. 2, Table S5). During the process of radicle elongation (from S2 to S3), the abundance of few metabolites changed. LysoPC 12:0 increased in all germination stages (S1, S2, S3), which may indicate that LysoPC may participate in the germination of *P. polyphylla*.

### Hormones of different classes changed variably during the seed germination of ***P. polyphylla***

Hormones play important roles in seed germination, and the roles of different plant hormones may vary during the process of *P. polyphylla* seed dormancy breaking. To investigate the role of plant hormones during the breaking of seed dormancy in *P. polyphylla*, hormones of 7 classes, ABA, gibberellin A (GA), auxin, jasmonic acid (JA), cytokinin (CK), salicylic acid (SA) and ethylene, were tested. These 7 hormone classes included 26 hormone molecules (Table S1). The content of most hormones, except ethylene, showed significant changes during the seed germination of *P. polyphylla* (Table 1). Significant changes in most hormones occurred at the stage of primary germination (S0 to S1). The total contents of ABA, CK, JA and SA decreased significantly at this stage, which decreased by 98.36%, 29.33%, 97.91%, and 51.38%, respectively (Table 1). However, the contents of GA and auxin increased by 147.31% and 91.68%, respectively, at this stage (Table 1). Only hormones in 3 classes changed from stage S1 to S2; JA and SA increased by 46.87% and 52.48%, respectively, while auxin decreased by 34.55% (Table 1). No significant change was observed in the total content of hormones from stage S2 to S3.

**Table 1:** Total content of each hormone class during the germination of *P. polyphylla* seeds

Hormone class	S0	S1	S2	S3	RC (%) S0 to S1
	Hormones content (ng/g)				
abscisic acid	42.70 ± 3.28^a^	0.70 ± 0.12^b^	0.63 ± 0.05^b^	0.62 ± 0.12^b^	-98.36
gibberellin A	5.20 ± 0.39^b^	12.86 ± 1.65^a^	12.89 ± 1.10^a^	12.06 ± 2.00^a^	147.31
auxin	31.62 ± 1.55^b^	60.61 ± 8.15^a^	39.67 ± 3.43^b^	32.41 ± 4.29^b^	91.68
jasmonic acid	11.08 ± 2.01^a^	7.83 ± 0.53^b^	11.50 ± 0.52^a^	9.30 ± 1.323^ab^	-29.33
cytokine	1.91 ± 0.29^a^	0.04 ± 0.00^b^	0.00 ± 0.00^b^	0.00 ± 0.00^b^	-97.91
salic acid	31.47 ± 3.35^a^	15.30 ± 1.60^c^	23.33 ± 1.37^b^	22.33 ± 1.88^b^	-51.38
ethylene	53.63 ± 9.03^a^	51.93 ± 0.86^a^	49.63 ± 8.13^a^	60.83 ± 1.18^a^	-3.17

The relative change (RC) in hormones from S0 to S1 is the percentage values for the difference between the values at S0 and S1 over the values at S0. Values in the same row with different letters are significantly different (*P* < 0.05). Values are means ± standard deviation (n = 3)

**Fig. 3:**
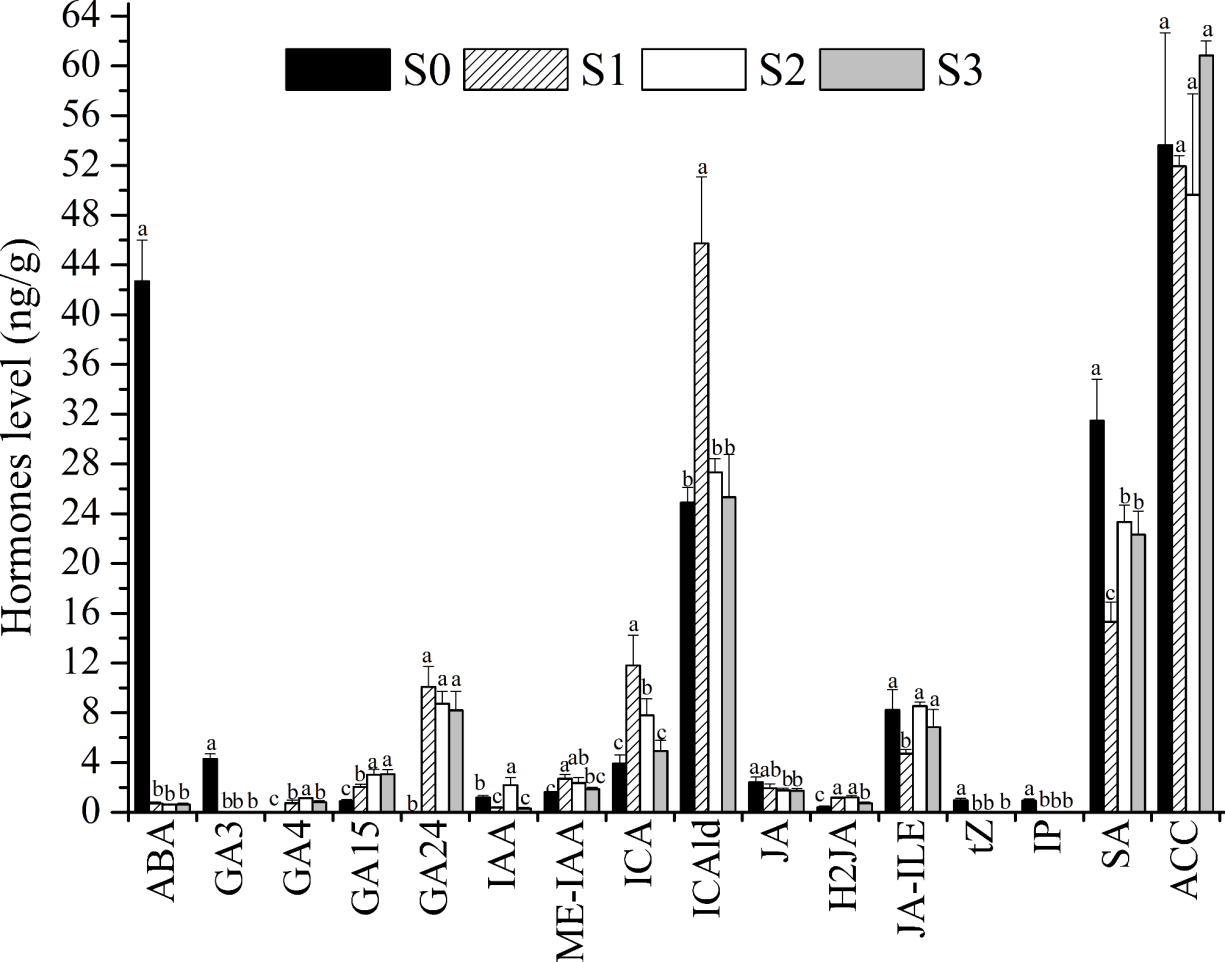
Changes in hormone levels during the germination of *P. polyphylla* seeds. The bars marked with different letters are significantly different from the others (*P* < 0.05). The values are means ± standard deviations (n = 3)

Among the five types of auxin we detected, the content of indole-3-acetic acid (IAA) decreased in the S1 stage and increased in the S2 stage, whereas the contents of methyl indole-3-acetate (ME-IAA), indole-3-carboxylic acid (ICA) and indole-3-carboxaldehyde (ICAld) increased in the S1 stage and decreased in the last two stages of S2 and S3, and we did not detect 3-indolebutyric acid (IBA) in *P. polyphylla* seeds (Fig. 3, Table S1). The metabolism of the three types of JA showed differences during different seed germination stages. JA showed little changes in content, but dihydrojasmonic acid (H2JA) increased significantly during the germination stage (S1, S2 and S3), while the content of jasmonoyl-L-isoleucine (JA-ILE) decreased greatly at the S1 stage and recovered to the level of the S0 stage in the last two stages of S2 and S3 (Fig. 3). Cytokinins such as trans-zeatin (tZ) and N6-isopentenyladenine (IP) were negatively correlated with the germination of *P. polyphylla* seeds and decreased to undetectable levels during the germination stage (S1, S2 and S3), whereas cis-zeatin (cZ) and dihydrozeatin (DZ) were not detected in *P. polyphylla* seeds throughout the whole germination stage (Fig. 3, Table S1). Taken together, these results may indicate that hormones of different classes and different hormone molecules play different roles during the seed germination of *P. polyphylla*: ABA, CK, and SA were negatively correlated with *P. polyphylla* seed germination, whereas GA and auxin were positively correlated with *P. polyphylla* seed germination.

### Non-13-hydroxylated GAs play important roles in the germination of ***Paris polyphylla***

GA was the most important hormone that was positively correlated with seed germination. GA_1_, GA_3_, GA_4_, and GA_7_ are the major bioactive GAs and are synthesized through two parallel pathways, namely, the 13-hydroxylation (13-OH) and non-13-hydroxylation (13-H) pathways. GA_1_ and GA_3_ are synthesized through the 13-OH pathway, while GA_4_ and GA_7_ are synthesized through the 13-H pathway [13]. The content of 13-OH GAs, GA_1_ and its precursors GA_53_, GA_19_, and GA_20_ were not detected, and the content of GA_3_ decreased dramatically to the level of the detection limit during the seed germination process (Fig. 3, Table S1). The content of 13-H GA, GA_4_ and its precursors GA_15_ and GA_24_ increased significantly during the germination process (Fig. 3). However, GA_9_, the precursor of GA_4_, was under the detection level limit in all the germination stages of *P. polyphylla* seeds (Table S1). Taken together, these results indicated that 13-H GAs played a positive role in *P. polyphylla* seed germination and that 13-OH GAs may play a negative role in *P. polyphylla* seed germination.

### DEGs identified during seed dormancy breaking of ***Paris polyphylla***

To evaluate the difference in gene expression during the seed dormancy breakdown of *P. polyphylla*, RNA sequencing (RNA-seq) was performed based on the Illumina HiSeq platform. A total of 110.19 Gb of clean bases were obtained, and the total amount of clean bases of all samples constituted more than 8 Gb. The sequencing error rate of each sample did not exceed 0.03%, and the Q30 value was greater than 93% (Table S6). The quality of the sequencing data was high enough for subsequent analysis. A total of 22,084 transcripts, with a mean length and N50 length of 1660 and 1867 nt, respectively, were assembled. The transcripts included those of 18,984 unigenes, and the mean length and N50 length of the unigenes were 1665 and 1880 nt, respectively. The square of the Pearson correlation coefficient (R^2^) between biological replicates was greater than 0.99, except for S0-1 with S0-3, the R^2^ of which was 0.98 (Fig. S3).

The DEGs were identified after transcriptome assembly and functionally annotated. To analyse the function of the assembled unigenes, the sequences of the unigenes were subjected to BLAST searches of seven public databases (i.e., Pfam, KEGG, NR, SwissProt, GO, KOG, and Trembl), and more than 90% of the genes were annotated in at least one database (Table S7). A total of 10,804 DEGs were identified in at least one of the three pairwise comparisons (i.e., S0 vs. S1, S1 vs. S2 and S2 vs. S3). The largest number of DEGs was found in S0 vs. S1 (6427 upregulated, 2445 downregulated). However, the smallest number of DEGs was found in S2 vs. S3, with only 203 and 536 genes up- and downregulated, respectively, and 614 DEGs were present in all three comparisons (Table S8, Fig. S4).

**Fig. 4:**
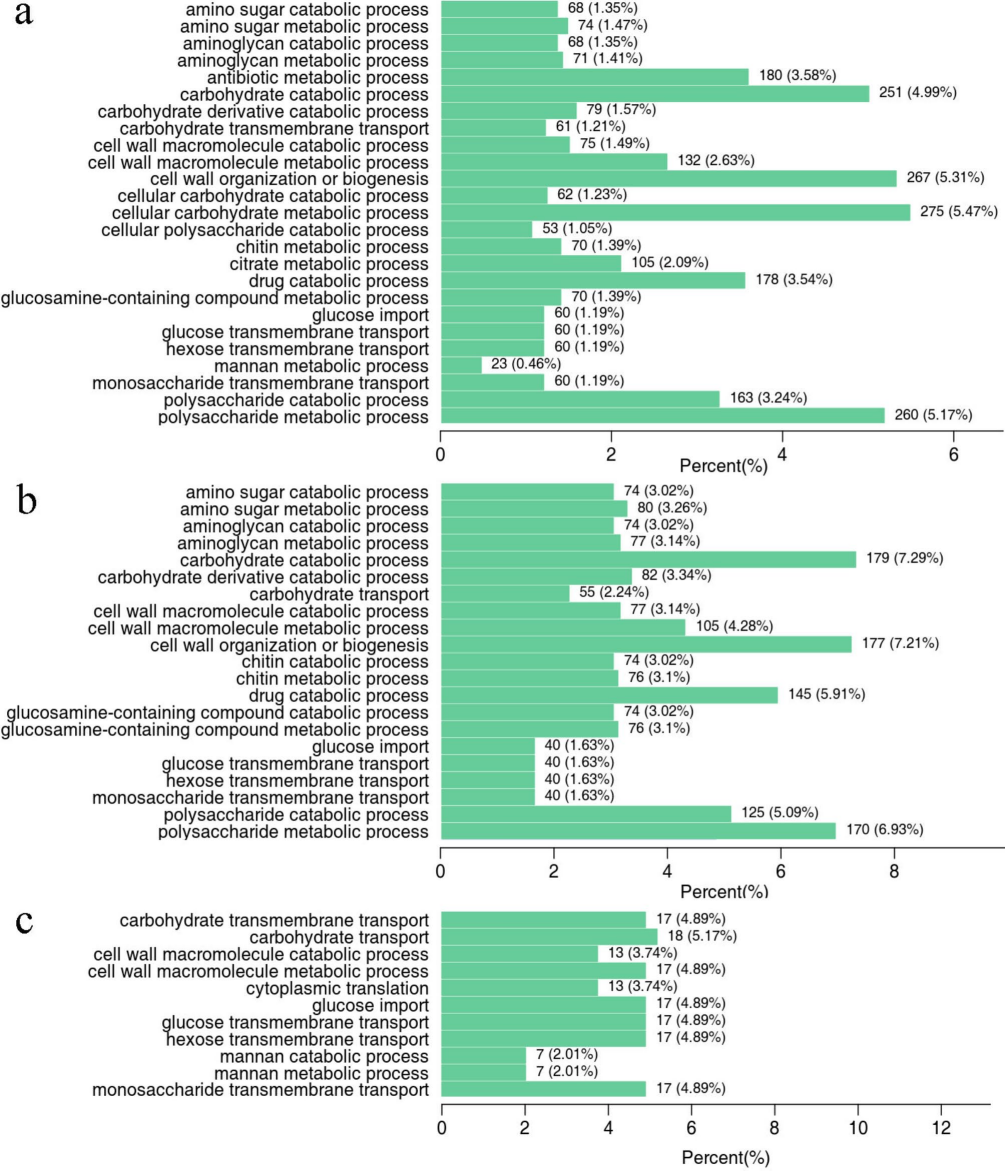
The top 50 GO enriched terms of DEGs related to biological processes between different comparisons. (**a**) S0 vs. S1, (**b**) S1 vs. S2, (**c**) S2 vs. S3

According to the GO annotation and classification results, the top 50 GO enriched terms were classified into three groups, biological processes, cellular components, and molecular functions, and plotted, the results of which are shown in Fig. 4 and S5. According to the GO enrichment results, 5909 DEGs were enriched in the primary germination stage (S0 vs. S1), 4430 DEGs were enriched in the radicle emergence stage (S1 vs. S2), and only 909 DEGs were enriched in the radicle elongation stage (S2 vs. S3). DEGs involved in biological processes were the most abundant in S0 vs. S1, and these DEGs were mainly associated with cellular carbohydrate metabolic processes, carbohydrate catabolic processes, polysaccharide metabolic processes, and cell wall organization or biogenesis (Fig. 4). The DEGs involved in cellular components were mainly associated with chromosomes, the apoplast, chromosomal parts, chromatin, and DNA packaging complexes in S0 vs. S1 (Fig. S5). Hydrolase activity, acting on glycosyl bonds and hydrolase activity, and hydrolysing O-glycosyl compounds were the main molecular functions of the enriched DEGs in S0 vs. S1. In the S1 vs. S2 and S2 vs. S3 comparisons, most DEGs were involved in molecular functions (Fig. S5), which is different from that in S0 vs. S1.

### Key genes related to ***P. polyphylla*** seed dormancy

**Fig. 5:**
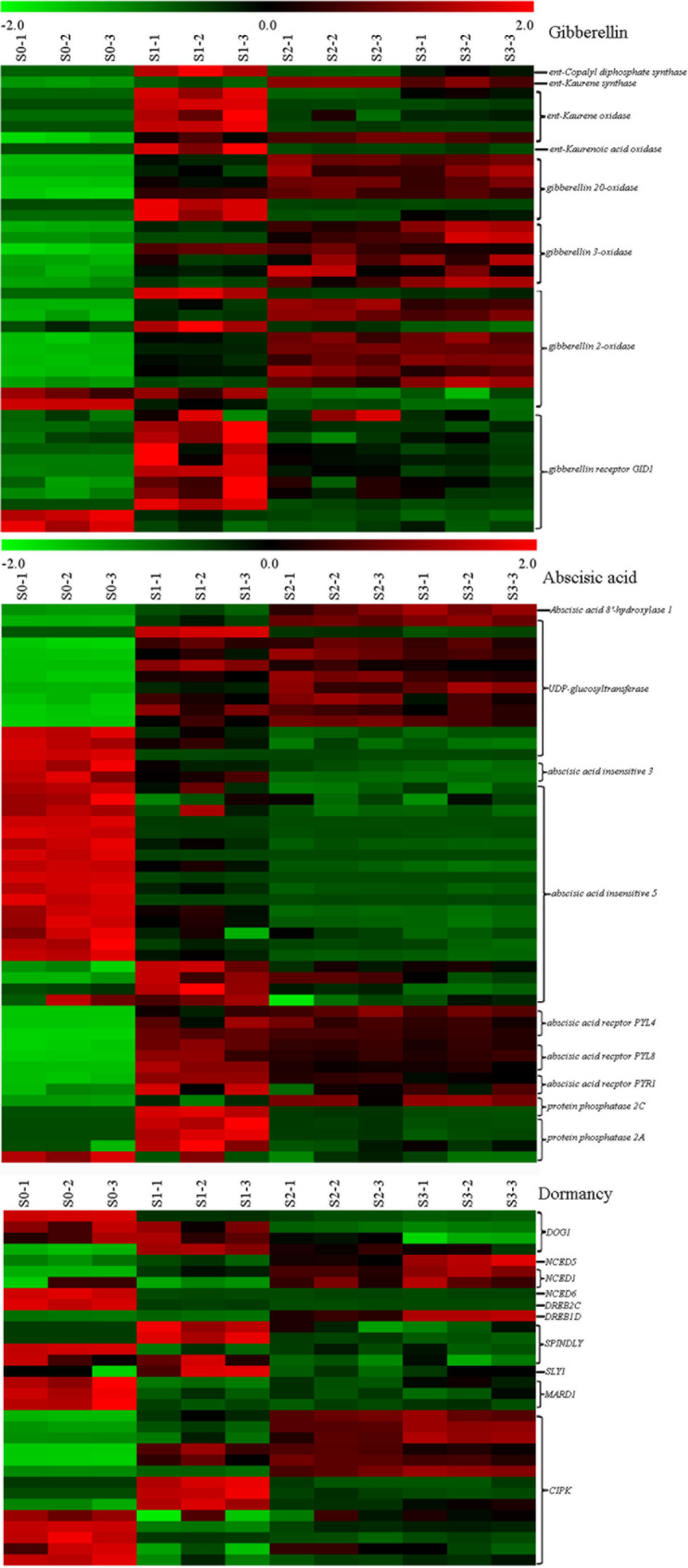
Hierarchal cluster of GA, ABA and dormancy -related DEGs in the different germination stages of *P. polyphylla*

The balance of GA and ABA is the main factor that controls the germination of seeds. According to the annotation results of the 7 public databases, we analysed GA-, ABA- and seed dormancy-related DEGs during different germination stages of *P. polyphylla*. Among the enzymes regulating GA synthesis, only one transcript of ent-copalyl diphosphate synthase (CPS) and ent-kaurene synthase (KS) was detected in *P. polyphylla* seeds, and these two genes were upregulated during seed germination (Fig. 5 and S6). Therefore, CPS and KS are encoded by a single gene in *P. polyphylla* seeds. Five *ent-kaurene oxidase* (*KO*) and one *ent-kaurenoic acid oxidase* (*KAO*) genes were found in *P. polyphylla* seeds, and these genes were upregulated during the primary germination S1 stage (Fig. 5 and S6). Gibberellin 13-oxidase is the key enzyme catalysing the synthesis of 13-OH GAs, and the transcript of the gene encoding gibberellin 13-oxidase was not detected in *P. polyphylla* seeds. Genes that are involved in the regulation of the synthesis of 13-H GA synthesis, such as *gibberellin 20-oxidase* and *gibberellin 3-oxidase*, were upregulated during the germination of *P. polyphylla* seeds. Fourteen transcripts of *gibberellin 2-oxidase*, a gibberellin deactivation-related gene, were detected in *P. polyphylla* seeds, 9 of which were upregulated, 2 of which were downregulated, and 3 of whose expression was not significantly changed. Nine transcripts were found to encode the gibberellin receptor GID1; these transcripts were upregulated during seed germination, and 2 of these transcripts were downregulated during this process (Fig. 5). Taken together, these results indicate that 13-H GAs may act in the dormancy breaking of *P. polyphylla* seeds.

ABA catalytic hydroxylation and conjugation are two methods of ABA catabolism. The expression of *CYP707A5*, which encodes abscisic acid 8’-hydroxylase 1, did not show significant changes at the S1 stage (Fig. 5 and S6). Most genes encoding UDP-glucosyltransferase (UGT), which can glucosylate ABA to its inactive form of glucose ester AGA-GE, were significantly upregulated, and some of them showed downregulation (Fig. 5 and S6). *ABA insensitive 3* and *5* (*ABI3*, *ABI5*), the key transcription factors that positively regulate seed dormancy, showed significant downregulation (Fig. 5 and S6). However, the expression of ABA receptor genes, such as *PYL4*, *PYL8* and *PYR1*, increased significantly during the germination of *P. polyphylla* seeds.

Genes positively related to dormancy, such as *DOG1*, *NCED6*, *DREB2C*, and *MARD1*, were downregulated, while genes negatively related to dormancy during the seed germination process, such as *SLY1*, were upregulated (Fig. 5 and S6). Two NCED genes, *NCED5* and *NCED6*, showed upregulation during this process. We identified 3 transcripts of *RGL1* in *P. polyphylla* seeds, whereas none of these 3 transcripts showed significant changes during seed germination. These results may indicate that part of the gene regulatory process of *P. polyphylla* seed dormancy may be the same as that of other plant species.

## Discussion

Breaking seed dormancy is a complicated process that involves remarkable reprogramming of genes expression, proteins, and metabolites [30–33]. Integrating transcriptomics and metabolomics is an effective way to study biological processes in plants [26, 28, 34]. Investigating the mechanism of seed dormancy at the molecular level is important for the breeding and cultivation of medicinal plants. In this study, using transcriptomics and metabolomics methods, we investigated different stages of seed dormancy breaking in *Paris polyphylla*, an important medicinal herbaceous species in China.

Changes in lipid metabolism-related genes were strongly correlated with *P. polyphylla* seed germination. Tang et al. found that genes encoding phospholipase D alpha 1, allene oxide cyclase, and long-chain acyl-CoA synthetase 2 were up-regulated, while genes encoding alpha-galactosidase were down-regulated during the germination of *P. polyphylla* seeds [3]. However, it is unclear whether the expression changes of these genes induced the changes of their metabolites. In our experiment, the most changed metabolites in the primary germination stage (S0) were lipids such as free fatty acids, glycerol ester, lysophosphatidylcholine (LPC), and lysophosphatidylethanolamine (LPE) (Table S5). The increase in free fatty acids may occur through the metabolism of storage lipids, and lipids can act as precursors of other lipids [35]. Lysophospholipids (lysoPLs) such as LPC and LPE are the products of phospholipid deacylation catalysed by phospholipid:diacylglycerol acyltransferase (PDAT) or phospholipase A (PLA) [36]. The expression of *PDAT* showed no significant difference among the four germination stages of *P. polyphylla*, while the expression of *PLA2* increased dramatically in the S1 stage (Fig. S6). The increase in LPC and LPE in *P. polyphylla* may be attributed to the PLA2-mediated deacylation of PC and PE. PLA2-induced generation of lysoPLs plays an important role in plant growth. LPC and LPE levels were shown to increased several times when plants are under stress [37]. lysoPLs are negatively correlated with the seed viability of soybean, a dicotyledonous species [38], whereas they are positively correlated with the seed viability of rice, a monocotyledonous species [39]. These results may indicate that PLA2-mediated lysoPL generation in the monocotyledonous *P. polyphylla* may have a positive effect on seed viability and may hasten seed germination.

**Fig. 6:**
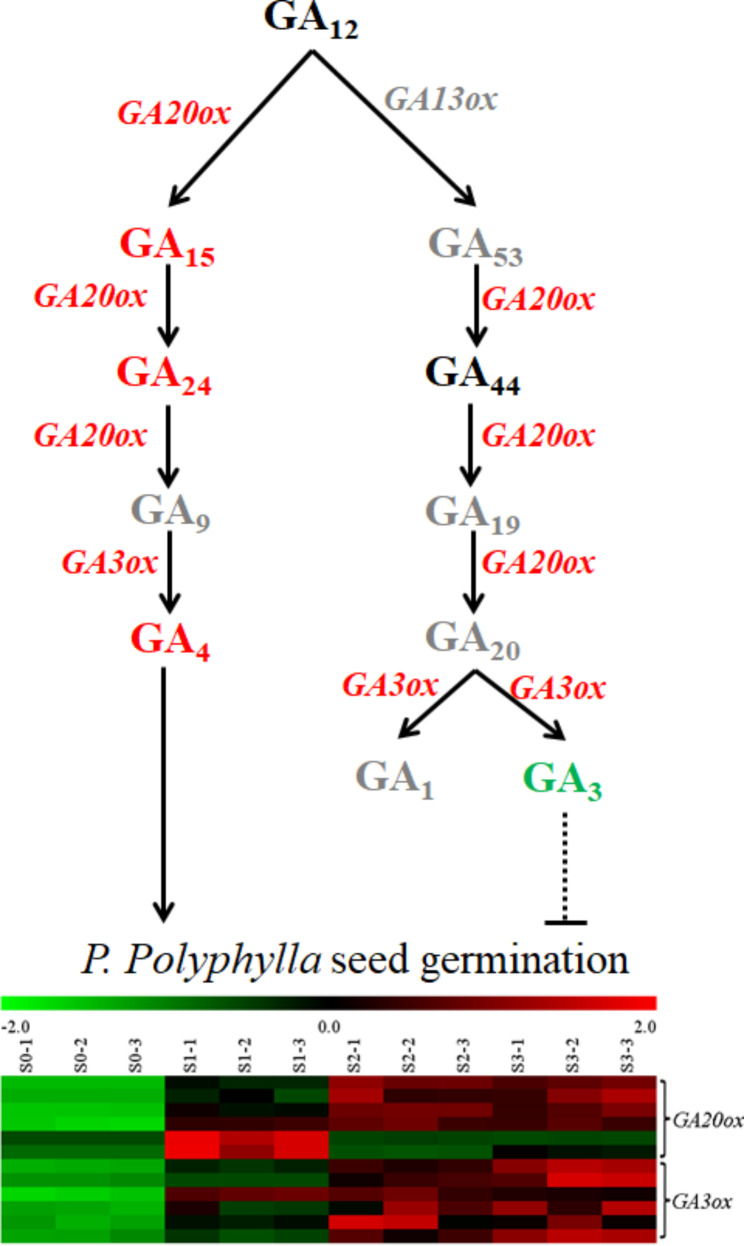
GAs metabolism and expression of genes related to GA synthesis during the seed dormancy breaking of *P. polyphylla*. Black font indicates that the GAs were not detected in our study. Red font indicates that the gene was upregulated and the content of GAs was increased during seed dormancy breaking of *P. polyphylla*. Grey font indicates that the transcript of the gene and GAs content were under the detection level limit. GA20ox catalysed 13-H GA synthesis pathway may play important roles in the seed dormancy breaking of *P. polyphylla*

The catabolism and signal transduction of ABA is important for seed germination. ABA catabolism occurs through two pathways: conversion to phaseic acid by cytochrome P_450_ monooxygenase (P_450_) encoded by *CYP707As* [10] or glycosylation by UGT to ABA-glucose ester (ABA-GE) [11]. It is reported that *CYP707A1*, *CYP707A4* and*UGT71B7* up-regulated at the beginning of germination [2], this may indicate that both of the two pathways of ABA catabolism participate in the germination of *P. polyphylla*. However, in our study, the expression of *CYP707A* was not synchronous with the change in ABA content during *P. polyphylla* seed germination (Figs. 4 and 6 and S3). The expression of most UGTs increased during germination, and the expression of some UGTs decreased during germination (Fig. 5 and S6). It is suspected that the decrease in ABA during the germination of *P. polyphylla* seeds may be attributed to the glycosylation process to form ABA-GE. *ABI3* and *ABI5* are two key transcription factors that play a role ABA signal transduction; the two transcripts of *ABI3* were downregulated, and 17 of 20 transcripts of *ABI5* were downregulated. The downstream transcription factor of ABI3, *NCED6*, was also downregulated. ABI3 may regulate the expression of NCED6 to control the dormancy of *P. polyphylla* seeds (Fig. 6 and S3). Therefore, ABA signal transduction during *P. polyphylla* seed germination may share the same pathways with those in other plants species [32].

The phytohormone GA is one of the most important hormones involved in seed germination. Both the increase in endogenous GAs and the application of exogenous GAs can break seed dormancy and accelerate seed germination. The conversion of geranylgeranyl diphosphate (GGDP) to *ent*-kaurene which catalyse by *ent*-copalyl diphosphate synthase (CPS) and *ent*-kaurene synthase (KS) is the first stage of GA biosynthesis [40]. CPS, as well as KS, is encoded by a single gene in most plant [13]. In *P. polyphylla* seeds, only one transcript of both CPS and KS was detected, which may indicate that CPS and KS were also encoded by a single gene in *P. polyphylla*. The up-regulation of these two genes may provide more precursors for GA biosynthesis during the dormancy breaking of *P. polyphylla* seeds. Among 136 fully characterized GA molecules, only GA_1_, GA_3_, GA_4_, and GA_7_ exhibit biological activity in plants [13]. GA_1_ and GA_3_ are synthesized through the 13-OH pathway, while GA_4_ and GA_7_ are synthesized through the 13-H pathway [13]. GA_1_ and GA_7_ were not detected in the *P. polyphylla* seeds. GA_3_ was detected only in the S0 stage but not in the other germination stages, while GA_4_ increased during *P. polyphylla* seed germination. GA 13-oxidase (GA13ox), which catalyses the synthesis of GA_12_ to GA_53_, is the key enzyme for the synthesis of 13-OH GAs, and the *CYP714* gene family encodes GA13ox [40]. We did not detect transcripts of *CYP714* in *P. polyphylla* seeds, which may cause the absence of GA13ox in seeds and, further, the absence of most 13-OH GAs, such as GA_53_, GA_19_, GA_20_, and GA_1_ (Table S1). GA_3_ was the only 13-OH GA that was present in ungerminated dry *P. polyphylla* seeds (S0 stage). GA_3_ may be synthesized in other tissues and transported into seeds during seed maturation of *P. polyphylla*. The synthesis of GA_4_ follows the order of GA_12_→GA_15_→GA_24_→GA_9_→GA_4_; GA20ox catalyses the metabolism of GA_12_ to GA_9_, and GA3ox catalyses GA_9_ to GA4 [15]. GA_15_ and GA_24_ increased during *P. polyphylla* seed germination, while GA_9_ was not detected (Table S1). Overexpression of *GA20ox* induced an increase in GA_4_ [12], and the increase in GA_4_ during *P. polyphylla* seed germination may be attributed to the significant up-regulation of *GA20ox* (Fig. S6). The synthesis of GA_4_ in *P. polyphylla* seeds may be different from that in other plant species, the synthesis of which does not occur through the catalysis of GA_9_ to GA_4_. To further confirm the different roles of exogenous GA_3_ and GA_4_ on *P. polyphylla* seed germination, we treated *P. polyphylla* seeds with 150 mg/mL GA_3_ and GA_4_ and found that after incubation for 3 months, only 15% of the GA_3_-treated seeds germinated, 30% of seeds with no treatment germinated, and 52% of GA_4_-treated seeds germinated. However, another study showed that the application of GA_3_ can also break seed dormancy in *P. polyphylla* [21]; the *P. polyphylla* seeds in our experiment were obtained from eastern Yunnan Province, while the seeds used by Zhou et al. were derived from central Yunnan Province. The different roles of GA_3_ in *P. polyphylla* seed germination may be attributed to the different growth environments of *P. polyphylla*. Thus, for *P. polyphylla* from eastern Yunnan Province, GA_3_ was negatively correlated with *P. polyphylla* seed germination, while GA_4_ was positively correlated with *P. polyphylla* seed germination.

The bioactivity of the various bioactive GAs is different; the bioactivity of GA_4_ is stronger than that of other GAs [13]. Magome et al. suggested that weakly active GAs may be involved in small growth change processes, whereas strongly active GAs would be useful for strong GA signal output, and that plants can dynamically adjust their use of strongly and weakly bioactive GAs [15]. The seed dormancy breaking process is an intense growth change that requires a strong GA signal, so GA_4_ with strong bioactivity is involved in the seed dormancy breaking of *P. polyphylla*; this same activity also occurs when *Syngonanthus verticillatus* seed dormancy is broken [18]. However, GA_1_ and GA_3_ increased dramatically, while GA_4_ and GA_7_ were not detected in polyaerial shoot rhizomes during *P. polyphylla* rhizome dormancy breaking, and GA_3_ application can also break rhizome dormancy in *P. polyphylla* [1]. Therefore, *P. polyphylla* has the ability to alter its bioactive GA synthesis to match GA-dependent growth.

## Conclusions

Through analysis of metabolomics and transcriptomics data, this study investigated the dormancy mechanism of eastern Yunnan *P. polyphylla* seeds. A decrease in ABA and increase in GA regulate the dormancy breaking of *P. polyphylla* seeds. The decrease in ABA was mainly attributed to the conversion of ABA to ABA-GE. *P. polyphylla* tends to block the synthesis of low-bioactivity 13-OH GAs (GA1 and GA3) and accelerate the synthesis of strong-bioactivity 13-H GA (GA_4_) during the process of breaking seed dormancy (Fig. 6). Different bioactive GAs act in different biological processes in *P. polyphylla*; GA_4_ may act in breaking seed dormancy, while GA_1_ and GA_3_ may act in breaking rhizome dormancy. Therefore, the choice of specific GA treatment in GA-dependent biological processes should be based on the habitats of *P. polyphylla* and the plant growth stage.

## Electronic supplementary material

Below is the link to the electronic supplementary material.

Supplementary Material 1

## Data Availability

The raw sequencing data has been submitted to NCBI (PRJNA939355, https://dataview.ncbi.nlm.nih.gov/object/PRJNA939355?reviewer=ege6n875uecv0e5jc643ocbmjl). All remaining are included in this published article and its supplementary information files.
