# Supplementary material for: Multiomics strategies for decoding seed dormancy breakdown in Paris polyphylla
Source: BMC Plant Biol. 2023 May 11;23:247. doi: 10.1186/s12870-023-04262-3 (PMC10173654; doi:10.1186/s12870-023-04262-3)
Supplement: Supplementary file 1 — Supplementary Material 1 [file 12870_2023_4262_MOESM1_ESM.docx]

**Multiomics strategies for decoding seed dormancy breakdown in *Paris polyphylla***

Guowei Zheng^1,#^, Wenchun Li^1,#^, Shunzhen Zhang ^1,#^, Qi Mi^1^, Wenxiu Luo^1^, Yanli Zhao1^1^, Xiangshi Qin^3^, Weijiao Li^1,*^, Shibiao Pu^1,*^, Furong Xu^2,*^

^1^College of Chinese Materia Medica, Yunnan University of Chinese Medicine, Kunming 650500, China

^2^College of Ethnic Medicines, Yunnan University of Chinese Medicine, Kunming 650500, China

^3^Germplasm Bank of Wild Species, Kunming Institute of Botany, Chinese Academy of Sciences, Kunming 650201, China

^#^ These authors contributed equally to this article.

^*^ To whom correspondence should be addressed. E-mail addresses:

Furong Xu, xfrong99@163.com; Weijiao Li, 460057707@qq.com; Shibiao Pu, 327834374@qq.com.

Supplementary Materials


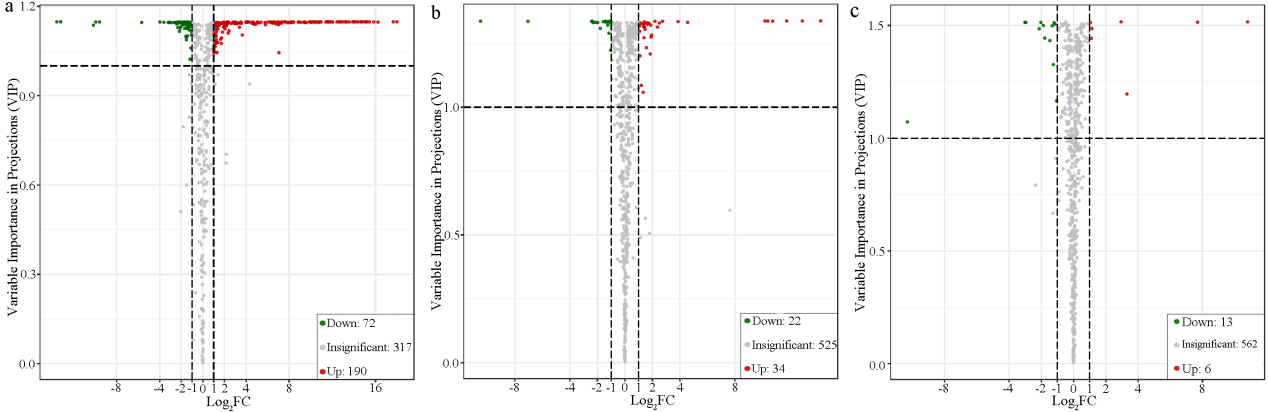


**Fig. S1.** Volcano plot of metabolites from (a) S0 vs S1, (b) S1vs S2, and (c) S2 vs S3.


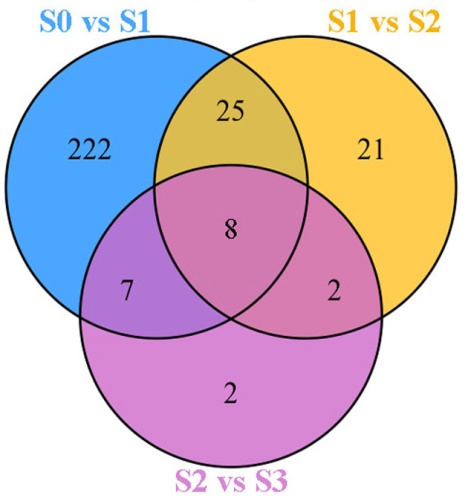


**Fig. S2.** Venn diagram showing the number of total SCMs.


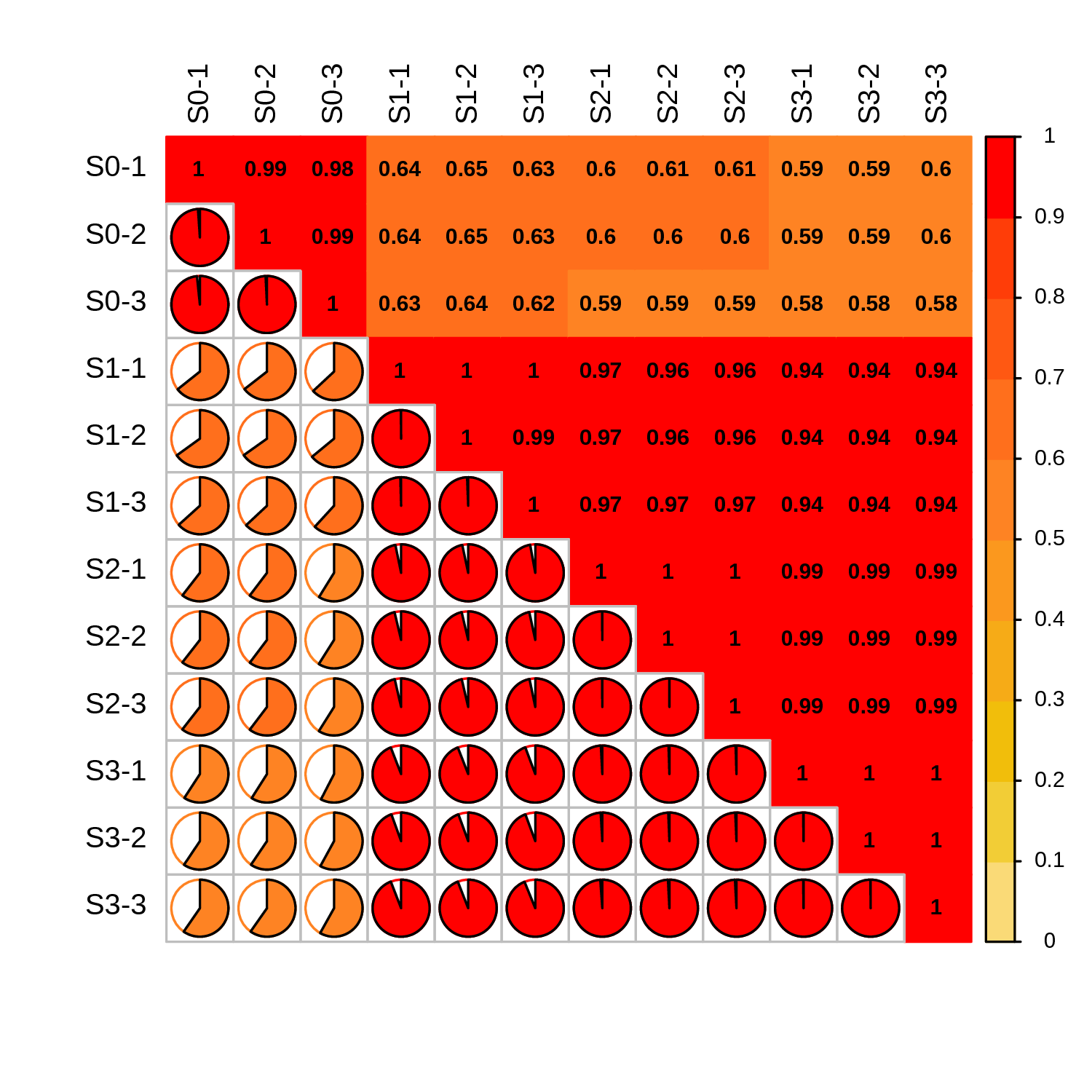


**Fig. S3.** The Pearson correlation coefficient heat map of different germination stage of *P. polyphylla* seeds.


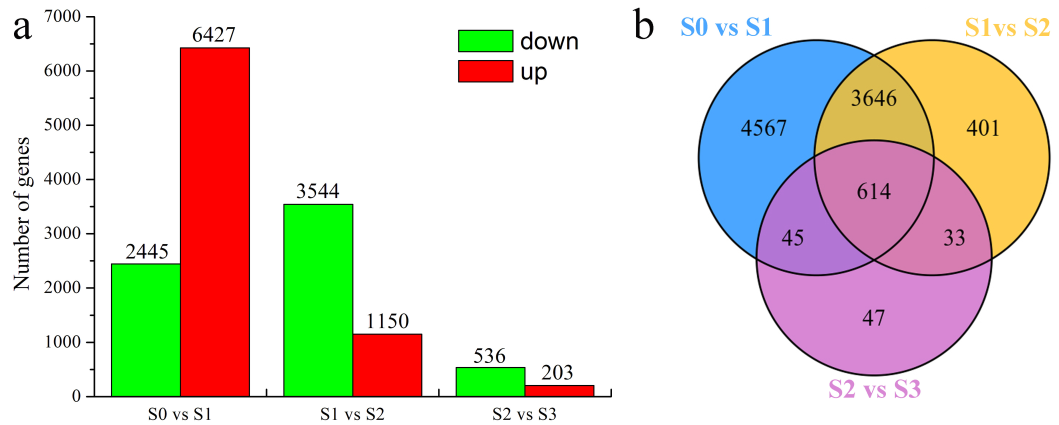


**Fig. S4.** Statistic of DEGs. (a) Number of genes differentially expressed in each germination stage. (b) Venn diagram of DEGs.


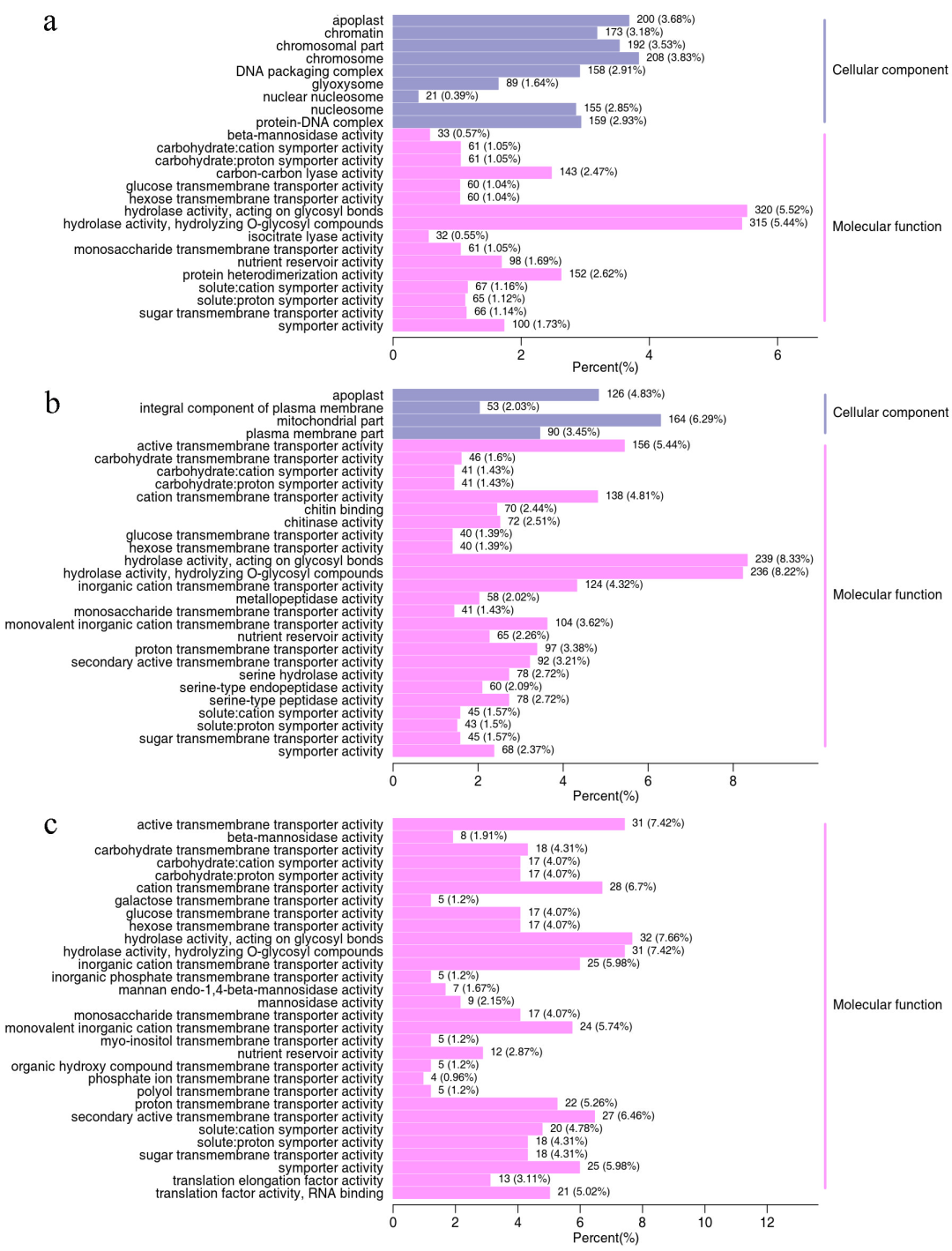


**Fig. S5.** GO classification of DEGs between different comparisons. (a) S0 vs. S1, (b) S1 vs. S2, (c) S2 vs. S3.





**Fig. S6.** Quantitative Q-RT PCR of transcripts related to P. polyphylla seed germination.

**Table S1.** Hormone changes during the germination of *P. polyphylla* seeds. N/A means the hormone were not detected in the seeds.

| Hormone class | Hormone | Abbreviation | S0 | S1 | S2 | S3 |
| --- | --- | --- | --- | --- | --- | --- |
|  |  |  | Hormone content (ng/g) | | | |
| Abscisic acid | Abscisic acid | ABA | 42.70 ± 3.28^a^ | 0.70 ± 0.12^b^ | 0.63 ± 0.05^b^ | 0.62 ± 0.12^b^ |
| Gibberellin | Gibberellin A_15_ | GA_15_ | 0.90 ± 0.14^c^ | 2.03 ± 0.24^b^ | 3.03 ± 0.43^a^ | 3.05 ± 0.38^a^ |
|  | Gibberellin A_24_ | GA_24_ | 0.00 ± 0.00^b^ | 10.08 ± 1.64^a^ | 8.72 ± 1.01^a^ | 8.20 ± 1.54^a^ |
|  | Gibberellin A_9_ | GA_9_ | N/A | N/A | N/A | N/A |
|  | Gibberellin A_4_ | GA_4_ | 0.00 ± 0.00^c^ | 0.75 ± 0.23^b^ | 1.14 ± 0.03^a^ | 0.81 ± 0.14^b^ |
|  | Gibberellin A_53_ | GA_53_ | N/A | N/A | N/A | N/A |
|  | Gibberellin A_19_ | GA_19_ | N/A | N/A | N/A | N/A |
|  | Gibberellin A_20_ | GA_20_ | N/A | N/A | N/A | N/A |
|  | Gibberellin A_1_ | GA_1_ | N/A | N/A | N/A | N/A |
|  | Gibberellin A_3_ | GA_3_ | 4.30 ± 0.40^a^ | 0.00 ± 0.00^b^ | 0.00 ± 0.00^b^ | 0.00 ± 0.00^b^ |
|  | Gibberellin A_7_ | GA_7_ | N/A | N/A | N/A | N/A |
| Auxin | Indole-3-acetic acid | IAA | 1.19 ± 0.16^b^ | 0.37 ± 0.06^c^ | 2.19 ± 0.63^a^ | 0.30 ± 0.09^c^ |
|  | Methyl indole-3-acetate | ME-IAA | 1.62 ± 0.06^c^ | 2.70 ± 0.35^a^ | 2.35 ± 0.45^ab^ | 1.86 ± 0.14^bc^ |
|  | 3-Indolebutyric acid | IBA | N/A | N/A | N/A | N/A |
|  | Indole-3-carboxaldehyde | ICAld | 24.90 ± 1.22^b^ | 45.73 ± 5.34^a^ | 27.33 ± 1.10^b^ | 25.33 ± 3.42^b^ |
|  | Indole-3-carboxylic acid | ICA | 3.92 ± 0.69^c^ | 11.81 ± 2.44^a^ | 7.80 ± 1.33^b^ | 4.92 ± 0.86^c^ |
| Jasmonic acid | Jasmonic acid | JA | 2.44 ± 0.40^a^ | 1.94 ± 0.34^ab^ | 1.76 ± 0.18^b^ | 1.73 ± 0.18^b^ |
|  | Jasmonoyl-L-Isoleucine | JA-ILE | 8.23 ± 1.62^a^ | 4.72 ± 0.33^b^ | 8.54 ± 0.31^a^ | 6.85 ± 1.43^a^ |
|  | Dihydrojasmonic acid | H_2_JA | 0.42 ± 0.09^c^ | 1.17 ± 0.05^a^ | 1.19 ± 0.15^a^ | 0.73 ± 0.09^b^ |
|  | Methyl jasmonate | MEJA | N/A | N/A | N/A | N/A |
| Cytokine | trans-Zeatin | tZ | 0.97 ± 0.15^a^ | 0.00 ± 0.00^b^ | 0.00 ± 0.00^b^ | 0.00 ± 0.00^b^ |
|  | cis-Zeatin | cZ | N/A | N/A | N/A | N/A |
|  | Dihydrozeatin | DZ | N/A | N/A | N/A | N/A |
|  | N6-Isopentenyladenine | IP | 0.94 ± 0.14^a^ | 0.04 ± 0.00^b^ | 0.00 ± 0.00^b^ | 0.00 ± 0.00^b^ |
| Salic acid | Salic acid | SA | 31.47 ± 3.35^a^ | 15.30 ± 1.60^c^ | 23.33 ± 1.37^b^ | 22.33 ± 1.88^b^ |
| Ethylene | 1-Amino-cyclopropane-1-carboxylic acid | ACC | 53.63 ± 9.03^a^ | 51.93 ± 0.86^a^ | 49.63 ± 8.13^a^ | 60.83 ± 1.18^a^ |

Values in the same row with different letters are significantly different (*P* < 0.05). Values are means ± standard deviation (n=3). N/A means the hormone were not detected in the seeds. N/A means the hormone were under the detection level limit.

**Table S2.** The detailed information of the hormones we tested.

| **Hormone class** | **Hormone** | **Abbreviaiton** | **Ionization model** | **Q1 (Da)** | **Q3 (Da)** | **Rt (min)** |
| --- | --- | --- | --- | --- | --- | --- |
| Auxin | Indole-3-acetic acid | IAA | [M+H]+ | 176.1 | 130.1 | 4.79 |
| Auxin | Methyl indole-3-acetate | ME-IAA | [M+H]+ | 190.1 | 130 | 5.83 |
| Auxin | 3-Indolebutyric acid | IBA | [M+H]+ | 204.3 | 130.2 | 5.48 |
| Auxin | Indole-3-carboxaldehyde | ICAld | [M+H]+ | 146.1 | 118 | 4.68 |
| Auxin | Indole-3-carboxylic acid | ICA | [M+H]- | 159.8 | 116.1 | 4.52 |
| CK | N6-Isopentenyladenine | IP | [M+H]+ | 204.25 | 136 | 3.94 |
| CK | trans-Zeatin | tZ | [M+H]+ | 220.2 | 136.1 | 3.09 |
| CK | cis-Zeatin | cZ | [M+H]+ | 220.25 | 136.1 | 3.2 |
| CK | Dihydrozeatin | DZ | [M+H]+ | 221.8 | 136.1 | 3.15 |
| JA | Methyl jasmonate | MEJA | [M+H]+ | 225.2 | 151.2 | 6.91 |
| JA | (+)-Jasmonic acid | JA | [M-H]- | 209.1 | 58.8 | 5.54 |
| JA | (+)-Dihydrojasmonic acid | H2JA | [M-H]- | 211.1 | 59 | 5.87 |
| JA | N-[(-)-Jasmonoyl]-(L)-Isoleucine | JA-ILE | [M-H]- | 322.401 | 129.7 | 6.02 |
| SA | Salicylic acid | SA | [M-H]- | 137 | 93 | 4.86 |
| ABA | (+)-cis,trans-Abscisic acid | ABA | [M-H]- | 263.1 | 153.1 | 5.03 |
| ETH | 1-Aminocyclopropanecarboxylic acid | ACC | [M+H]+ | 102.2 | 56 | 0.78 |
| GA | Gibberellin A1 | GA1 | [M-H]- | 347 | 259 | 4.24 |
| GA | Gibberellin A3 | GA3 | [M-H]- | 345 | 239 | 4.2 |
| GA | Gibberellin A4 | GA4 | [M-H]- | 331 | 212.8 | 5.84 |
| GA | Gibberellin A7 | GA7 | [M-H]- | 329.2 | 223 | 5.77 |
| GA | Gibberellin A9 | GA9 | [M-H]- | 315.1 | 270.9 | 6.78 |
| GA | Gibberellin A15 | GA15 | [M-H]- | 329.1 | 131 | 6.76 |
| GA | Gibberellin A19 | GA19 | [M-H]- | 361.2 | 273.2 | 4.87 |
| GA | Gibberellin A20 | GA20 | [M-H]- | 331.3 | 287 | 5.07 |
| GA | Gibberellin A24 | GA24 | [M-H]- | 345.1 | 257.1 | 6.01 |
| GA | Gibberellin A53 | GA53 | [M-H]- | 347.1 | 303.1 | 5.42 |

**Table S3**. The primer sequence used in the Q-PCR

| ID | Gene |  | sequence(5'-3') | product size |
| --- | --- | --- | --- | --- |
| A20012988a_transcript_10096 | *TUA* | F | TCGACGGTGCGCTTAATGT | 174bp |
|  |  | R | TCATGGAGGACGGCTCAAAG |  |
| A20012988a_transcript_1414 | *CPS（ent-copalyl diphosphate synthase）* | F | ACTCCAATGCTCTACTGGCG | 177bp |
|  |  | R | CGACCAACGCTTCAACCAAG |  |
| A20012988a_transcript_2944 | *KS（ent-kaurene synthase）* | F | TGAATGGCTTCGCGTACAGA | 101bp |
|  |  | R | AGGCCGTCGCAAGGAAATAG |  |
| A20012988a_transcript_8560 | *ent-kaurene oxidase（KO）* | F | GACCAGTTCTACTCGGCTCC | 176bp |
|  |  | R | CTCCCGCATTCGCAAGAAAC |  |
| A20012988a_transcript_12352 | *ent-kaurenoic acid oxidase(KAO)* | F | TTCTTGCTTCTGGTCGCTGG | 116bp |
|  |  | R | GCAAGGGATGGGTTGGTCTT |  |
| A20012988a_transcript_15117 | *GA20ox5* | F | GTGGACCAACGACCAGTACC | 101bp |
|  |  | R | AGTCAGGGTTGCCGTTGAAA |  |
| A20012988a_transcript_14507 | *GA20ox* | F | CTAGTAAGCGAGGAGCACCC | 193bp |
|  |  | R | TGTCCCTTGAGCTGAGTCCA |  |
| A20012988a_transcript_15659 | *GA3ox* | F | GCGGATTTCGCAGTACAAGG | 118bp |
|  |  | R | GTGCACCTCGAGTCCCTCTA |  |
| A20012988a_transcript_12775 | *GA3ox* | F | AGAAAGGGATGGCCGACTTG | 159bp |
|  |  | R | CAGGGGAGGTGGAATCTTCG |  |
| A20012988a_transcript_15429 | *GA2ox* | F | TAACCCAGCACACCGATAGC | 103bp |
|  |  | R | TTTGGCTTCACGGAGTTCCA |  |
| A20012988a_transcript_5697 | *CYP707A5* | F | GGATGTCGAGTACGAGGGGT | 192bp |
|  |  | R | AACTCATTTCCAGGGCACGA |  |
| A20012988a_transcript_7135 | *UGT* | F | GCTGTTATGGGCGAGGTTCT | 138bp |
|  |  | R | CTGGATTGGCGAGGCACTTA |  |
| A20012988a_transcript_10704 | *UGT85A24* | F | GGGATGCACCGAGTGGTTAG | 195bp |
|  |  | R | GAGAACTCGGATGGCACGAC |  |
| A20012988a_transcript_19220 | *ABI3* | F | TGCCGGTTCTATCTGTCAAGG | 181bp |
|  |  | R | AGAGCGATCATATCTCCGCA |  |
| A20012988a_transcript_20967 | *PP2C* | F | GGGTGATATACTGGGACGGC | 151bp |
|  |  | R | CTCGCCAGTACGAGACACTC |  |
| A20012988a_transcript_9637 | *PP2A* | F | GGCTTGACCTTGATTGCTCG | 103bp |
|  |  | R | AGCAGTAGTTGGGTGCTGAG |  |
| A20012988a_transcript_18675 | *DOG1* | F | CCTCGCTCACTACCACGAGTA | 110bp |
|  |  | R | AGAGGAAGGTGCGCTCGTAG |  |
| A20012988a_transcript_7025 | *NCED6* | F | GGTATTGCCGAAGCATGACG | 165bp |
|  |  | R | GAAAACATTGCGTCGGGAGG |  |
| A20012988a_transcript_20348 | *DREB2C* | F | CCCGAGAACCAGTCATGCG | 115bp |
|  |  | R | AGGTGAATGTGCCGAGCCA |  |
| A20012988a_transcript_4024 | *SLY1* | F | AATGCCACCTCCTAGTGCAG | 111bp |
|  |  | R | TCGCTGAACCCTTGTCTTCG |  |
| A20012988a_transcript_12126 | *LPCAT* | F | CAGTGATTCTAAGGTGCGGC | 198bp |
|  |  | R | ACTCGCTTTTCAGGCAGACC |  |
|  |  | R | CATGGCGCACCCTTTGTTC |  |
| A20012988a_transcript_11952 | *PLA2* | F | TCCGGTTCTTCCCGTATCCT | 117bp |
|  |  | R | GTGAAGGTAGGTCTCGCACC |  |

**Table S4.** The number of metabolites tested and the percentage of their peak area during the germination of *P. polyphylla* seeds.

| First Class of metabolites | Detected  Number | Percentage of total peak area (%) | | | |
| --- | --- | --- | --- | --- | --- |
|  |  | S0 | S1 | S2 | S3 |
| Amino acids and derivatives | 86 | 18.99 ± 0.20^b^ | 16.58 ± 0.52^c^ | 19.15 ± 0.52^b^ | 20.66 ± 0.41^a^ |
| Organic acids | 62 | 18.06 ± 0.36^a^ | 7.29 ± 0.09^d^ | 9.13 ± 0.14^c^ | 9.65 ± 0.16^b^ |
| Lipids | 107 | 16.93 ± 2.20^c^ | 50.41 ± 1.37^a^ | 44.91 ± 0.88^b^ | 43.42 ± 0.96^b^ |
| Nucleotides and derivatives | 42 | 9.40 ± 0.23^a^ | 3.02 ± 0.08^c^ | 3.38 ± 0.08^b^ | 3.13 ± 0.03^c^ |
| Phenolic acids | 83 | 9.19 ± 0.90^a^ | 5.67 ± 0.15^b^ | 5.62 ± 0.25^b^ | 5.24 ± 0.11^b^ |
| Alkaloids | 39 | 4.12 ± 0.03^b^ | 3.91 ± 0.15^c^ | 4.44 ± 0.09^a^ | 4.55 ± 0.03^a^ |
| Steroids | 23 | 2.82 ± 0.12^a^ | 1.84 ± 0.06^b^ | 1.82 ± 0.07^b^ | 1.70 ± 0.04^b^  70 |
| Terpenoids | 9 | 1.43 ± 0.18^a^ | 0.85 ± 0.12^b^ | 0.81 ± 0.02^b^ | 0.74 ± 0.05^b^ |
| Flavonoids | 39 | 1.32 ± 0.03^a^ | 0.59 ± 0.02^c^ | 0.78 ± 0.02^b^ | 0.82 ± 0.02^b^ |
| Lignans and Coumarins | 13 | 0.13 ± 0.01^a^ | 0.10 ± 0.01^bc^ | 0.11 ± 0.00^b^ | 0.09 ± 0.01^c^ |
| Tannins | 4 | 0.06 ± 0.01^a^ | 0.02 ± 0.00^b^ | 0.02 ± 0.00^b^ | 0.02 ± 0.00^b^ |
| Quinones | 1 | 0.00 ± 0.00^a^ | 0.00 ± 0.00^a^ | 0.00 ± 0.00^a^ | 0.00 ± 0.00_a_ |
| Others | 77 | 17.55 ± 1.32^a^ | 9.71 ± 0.39^b^ | 9.82 ± 0.20^b^ | 9.97 ± 0.40^b^ |

Table S5. Changes of SCMs during *P. polyphylla* seed germination.

| Metabolites | S0_vs_S1 | S1_vs_S2 | S2_vs_S3 |
| --- | --- | --- | --- |
| 1-methylguanidine | up | FALSE | FALSE |
| L-Alanine* | down | FALSE | FALSE |
| N-Methylglycine* | down | FALSE | FALSE |
| Succinic anhydride | down | FALSE | FALSE |
| Cadaverine | FALSE | FALSE | down |
| Hydroxypyruvic acid | down | FALSE | FALSE |
| 3-Hydroxybutyric acid | down | FALSE | FALSE |
| Malonic acid | down | FALSE | FALSE |
| Indole | up | FALSE | FALSE |
| Betaine | up | FALSE | FALSE |
| Succinic acid | down | FALSE | FALSE |
| Aminomalonic acid | down | FALSE | FALSE |
| Phenethylamine | up | down | FALSE |
| Nicotinamide | up | FALSE | FALSE |
| 2-Aminoethanesulfonic acid | up | FALSE | FALSE |
| Thymine | up | FALSE | down |
| 5-Oxo-L-Proline | FALSE | up | FALSE |
| Cycloleucine | up | FALSE | FALSE |
| N-Propionylglycine | up | FALSE | FALSE |
| Ethylmalonic acid* | down | down | up |
| Glutaric acid* | down | FALSE | FALSE |
| L-Ornithine | up | FALSE | FALSE |
| L-Aspartic Acid | up | FALSE | FALSE |
| Adenine | down | FALSE | FALSE |
| Phenylethanolamine* | FALSE | up | up |
| L-Tyramine* | FALSE | up | up |
| 3-Hydroxybenzoic Acid | down | up | FALSE |
| Muconic acid | up | FALSE | FALSE |
| 1-Methylpiperidine-2-carboxylic acid | up | down | down |
| 2,3-Dimethylsuccinic acid | up | FALSE | FALSE |
| 4-Acetamidobutyric acid | down | FALSE | FALSE |
| Adipic Acid* | down | up | FALSE |
| (S)-2-Acetyl-2-Hydroxybutanoic Acid* | FALSE | up | FALSE |
| 2,2-Dimethylsuccinic acid | up | FALSE | FALSE |
| (R)-3-Hydroxy-3-Methyl-2-Oxopentanoic Acid* | up | FALSE | FALSE |
| L-Glutamine | up | FALSE | FALSE |
| L-Lysine | up | FALSE | FALSE |
| L-threo-3-Methylaspartate* | down | FALSE | FALSE |
| Cinnamic acid | down | FALSE | FALSE |
| D-(-)-Arabinose* | up | FALSE | FALSE |
| Xylitol* | up | FALSE | FALSE |
| D-Arabitol* | up | FALSE | FALSE |
| Ribitol* | up | FALSE | FALSE |
| L-Histidine | up | FALSE | FALSE |
| 3-Indoleacetonitrile | up | FALSE | FALSE |
| 5-Acetamidopentanoic Acid | up | FALSE | FALSE |
| 3-Hydroxyphenylacetic Acid Methyl Ester | up | up | down |
| 3-(3-Hydroxyphenyl)-propionate acid | up | up | down |
| Gallacetophenone | up | FALSE | FALSE |
| Pyridoxine | down | FALSE | FALSE |
| L-Glycyl-L-proline | FALSE | up | FALSE |
| 9-Oxononanoic acid | down | FALSE | FALSE |
| Shikimic acid | FALSE | up | FALSE |
| L-Ascorbic acid | down | FALSE | FALSE |
| 2-Isopropylmalic Acid | down | FALSE | FALSE |
| 4-Methoxycinnamic acid | up | FALSE | FALSE |
| Caffeic acid | down | FALSE | FALSE |
| Dihydroactinidiolide | down | FALSE | FALSE |
| D-Sorbitol* | up | FALSE | FALSE |
| Dulcitol* | up | FALSE | FALSE |
| Mannitol* | up | FALSE | FALSE |
| 3-O-Methylgallic Acid | FALSE | FALSE | down |
| 3-Phospho-D-glyceric acid | up | FALSE | FALSE |
| N6-Acetyl-L-lysine | up | FALSE | FALSE |
| N-Acetyl-L-glutamic acid | down | up | FALSE |
| 2,6-Diaminooimelic acid | up | FALSE | FALSE |
| N-Acetyl-L-methionine | up | FALSE | FALSE |
| Dimethyl Phthalate | up | FALSE | FALSE |
| Acetryptine | up | down | FALSE |
| L-Tryptophan | up | FALSE | FALSE |
| 4,8-Dihydroxyquinoline-2-carboxylic acid | up | FALSE | FALSE |
| Methoxyindoleacetic acid | up | FALSE | FALSE |
| 5,7-Dimethoxycoumarin (Limettin)(Citropten) | down | FALSE | FALSE |
| N-Acetyl-L-phenylalanine | up | FALSE | FALSE |
| 3-[(1-Carboxyvinyl)oxy]benzoic acid | up | FALSE | FALSE |
| Methyl ferulate | down | FALSE | FALSE |
| N-Acetyl-L-Arginine | up | FALSE | FALSE |
| D-Pantothenic Acid | down | FALSE | FALSE |
| 6,7-Dimethoxy-4-methylcoumarin | up | FALSE | FALSE |
| N-Acetyl-D-mannosamine* | up | FALSE | FALSE |
| L-Glycyl-L-phenylalanine | down | FALSE | FALSE |
| Dehydrovomifoliol | down | FALSE | FALSE |
| N-Acetyl-L-tyrosine | FALSE | up | FALSE |
| Vomifoliol | down | FALSE | FALSE |
| 2'-Deoxycytidine | down | FALSE | FALSE |
| Myristic Acid | up | FALSE | FALSE |
| p-Coumaroylputrescine | FALSE | up | FALSE |
| L-Lysine-Butanoic Acid | up | FALSE | down |
| L-Alanyl-L-Phenylalanine | up | FALSE | FALSE |
| 1-O-p-Cumaroylglycerol | up | FALSE | FALSE |
| Thymidine | down | up | down |
| Pentadecanoic Acid | up | FALSE | FALSE |
| Cytidine | down | FALSE | FALSE |
| Uridine | down | FALSE | FALSE |
| Biotin | down | FALSE | FALSE |
| N-Acetyl-L-Tryptophan | up | FALSE | FALSE |
| Pyridoxine-5'-phosphate | up | FALSE | FALSE |
| γ-Glu-Cys | down | FALSE | FALSE |
| 2'-Deoxyadenosine | down | up | FALSE |
| 5-Methyluridine | down | FALSE | FALSE |
| D-Fructose 6-phosphate | down | FALSE | FALSE |
| D-Glucose 6-phosphate | down | FALSE | FALSE |
| Adenosine | down | FALSE | FALSE |
| 2'-Deoxyguanosine | down | FALSE | FALSE |
| 9-(Arabinosyl)hypoxanthine | down | FALSE | FALSE |
| Cis-10-Heptadecenoic Acid | up | FALSE | FALSE |
| Emodin | up | FALSE | FALSE |
| Heptadecanoic acid | up | FALSE | FALSE |
| 16-Hydroxyhexadecanoic acid | up | FALSE | FALSE |
| 2-Hydroxyhexadecanoic acid | up | FALSE | FALSE |
| L-Saccharopine | up | FALSE | FALSE |
| Pantetheine | up | up | FALSE |
| γ-Linolenic Acid* | up | FALSE | FALSE |
| Crepenynic acid | up | FALSE | FALSE |
| α-Linolenic Acid* | up | FALSE | FALSE |
| (9Z,11E)-Octadecadienoic acid* | up | FALSE | FALSE |
| Linoleic acid* | up | FALSE | FALSE |
| 11-Octadecanoic acid(Vaccenic acid) | up | FALSE | FALSE |
| Guanosine | down | FALSE | FALSE |
| Xanthosine | down | down | FALSE |
| Gastrodin | up | FALSE | FALSE |
| Argininosuccinic acid | down | FALSE | FALSE |
| 13S-Hydroxy-9Z,11E,15Z-octadecatrienoic acid | down | FALSE | FALSE |
| 13(S)-HODE;13(S)-Hydroxyoctadeca-9Z,11E-dienoic acid* | up | FALSE | FALSE |
| 9(10)-EpOME;(9R,10S)-(12Z)-9,10-Epoxyoctadecenoic acid* | up | FALSE | FALSE |
| 9S-Hydroxy-10E,12Z-octadecadienoic acid* | up | FALSE | FALSE |
| N7-Methylguanosine | down | FALSE | FALSE |
| 9,10-Epoxyoctadecanoic Acid | up | FALSE | FALSE |
| 1-O-Salicyl-D-glucose | down | FALSE | FALSE |
| Salicylic acid-2-O-glucoside | FALSE | up | FALSE |
| 2R-Hydroxyoctadecanoic Acid | up | FALSE | FALSE |
| 4-O-Glucosyl-3,4-dihydroxybenzyl alcohol | up | FALSE | FALSE |
| Dihydroquercetin(Taxifolin) | up | FALSE | FALSE |
| Eicosadienoic acid | up | FALSE | FALSE |
| 2-(D-Glucosyloxy)-4-hydroxybenzeneacetonitrile | up | FALSE | FALSE |
| 2-(Dimethylamino)guanosine | down | FALSE | FALSE |
| 13S-Hydroperoxy-9Z,11E-octadecadienoic acid | up | FALSE | FALSE |
| Arachidic acid | up | FALSE | FALSE |
| Cafestol | up | down | FALSE |
| Uridine 5'-monophosphate | down | FALSE | FALSE |
| N-Oleoylethanolamine | down | FALSE | FALSE |
| 6-Hydroxy-5,7,4'-trimethoxyflavone | down | down | up |
| Cyclic 3',5'-Adenylic acid | down | FALSE | FALSE |
| 2-Amino-1,3-eicosanediol | up | FALSE | FALSE |
| 1-O-Vanilloyl-D-Glucose | down | FALSE | FALSE |
| 15-Hydroperoxyicosatetraenoic acid | up | FALSE | FALSE |
| O-Feruloyl 4-hydroxycoumarin | up | FALSE | FALSE |
| 1-O-p-Coumaroylquinic acid | FALSE | up | FALSE |
| 4-Pyridoxic acid-O-glucoside | up | FALSE | FALSE |
| Adenosine 5'-monophosphate | down | FALSE | FALSE |
| 3'-Adenylic Acid | down | FALSE | down |
| 1-Stearidonoyl-Glycerol | up | FALSE | FALSE |
| 1-Methoxyphaseollin | up | down | FALSE |
| 2-α-Linolenoyl-glycerol | up | FALSE | FALSE |
| 1-Linoleoylglycerol* | up | FALSE | FALSE |
| 2-Linoleoylglycerol* | up | FALSE | FALSE |
| 1-Monolinolein | up | FALSE | FALSE |
| Piperitol | down | FALSE | FALSE |
| 1-Oleoyl-Sn-Glycerol | up | FALSE | FALSE |
| Guanosine 5'-monophosphate | down | FALSE | FALSE |
| Syringin | up | FALSE | FALSE |
| Riboflavin | up | FALSE | FALSE |
| Regaloside H | FALSE | up | FALSE |
| Benzyl-(2''-O-xylosyl)glucoside | FALSE | up | FALSE |
| Uridine 5'-diphosphate | down | FALSE | FALSE |
| 2,3,5,4'-Tetrahydroxystilbene-2-O-D-glucoside | down | FALSE | FALSE |
| Piceatannol-3'-O-glucoside | up | FALSE | FALSE |
| Ebeinone | up | FALSE | FALSE |
| Diosgenin | up | down | down |
| Regaloside L | up | FALSE | FALSE |
| Trehalose 6-phosphate | down | FALSE | FALSE |
| LysoPE 14:0 | up | FALSE | FALSE |
| Taraxerol | up | FALSE | FALSE |
| Ergosterol peroxide | up | FALSE | FALSE |
| Pennogenin | up | FALSE | FALSE |
| Schisandrol A | up | FALSE | down |
| 6-O-Caffeoylarbutin | down | FALSE | FALSE |
| Dihydrocharcone-4'-O-glucoside | up | FALSE | FALSE |
| LysoPE 15:0 | up | up | FALSE |
| LysoPE 15:0(2n isomer) | up | down | FALSE |
| LysoPC 12:0 | up | up | up |
| 24-hydroxy Pennogenin | up | FALSE | FALSE |
| L-Glutaminyl-L-valyl-L-valyl-L-cysteine | down | FALSE | FALSE |
| Luteolin-4'-O-glucoside | down | FALSE | FALSE |
| Kaempferol-3-O-galactoside (Trifolin)* | down | FALSE | FALSE |
| LysoPE 16:1* | up | FALSE | FALSE |
| LysoPE 16:1(2n isomer)* | up | down | FALSE |
| Sieboldin | down | FALSE | FALSE |
| LysoPE 16:0* | up | FALSE | FALSE |
| LysoPE 16:0(2n isomer) | up | FALSE | FALSE |
| Coumarin O-rutinoside | up | FALSE | FALSE |
| 24,30-Dihydroxy-12(13)-enolupinol | up | FALSE | FALSE |
| Sibiricose A3 | up | FALSE | FALSE |
| LysoPE 17:1* | up | FALSE | FALSE |
| LysoPE 17:1(2n isomer)* | up | down | FALSE |
| LysoPC 14:0 | up | FALSE | FALSE |
| 10-Formyltetrahydrofuran | down | FALSE | FALSE |
| LysoPE 18:3* | up | FALSE | FALSE |
| LysoPE 18:3(2n isomer)* | up | FALSE | FALSE |
| LysoPE 18:2(2n isomer)* | up | FALSE | FALSE |
| LysoPE 18:2* | up | FALSE | FALSE |
| Isorhamnetin-7-O-glucoside (Brassicin) | down | FALSE | FALSE |
| LysoPE 18:1 | up | FALSE | FALSE |
| LysoPC 15:1 | up | FALSE | FALSE |
| LysoPE 18:0(2n isomer) | up | down | FALSE |
| LysoPE 18:0 | up | FALSE | FALSE |
| LysoPC 15:0(2n isomer)* | up | FALSE | FALSE |
| LysoPC 15:0* | up | FALSE | FALSE |
| 5-O-p-Coumaroylshikimic acid O-glucoside | up | FALSE | FALSE |
| 4-Hydroxycoumarin di-glucoside | up | FALSE | FALSE |
| 27,28-Dicarboxyl ursolic acid | up | FALSE | FALSE |
| Feruloylcaffeoyltartaric acid | up | FALSE | FALSE |
| Cistanoside F | up | FALSE | FALSE |
| 3-Hydroxy-4-isopropylbenzylalcohol-3-O-sophoroside | up | FALSE | FALSE |
| Cimicifugamide A | up | FALSE | down |
| LysoPC 16:2 | up | FALSE | FALSE |
| LysoPC 16:2(2n isomer) | up | FALSE | FALSE |
| LysoPC 16:1(2n isomer)* | up | FALSE | FALSE |
| LysoPC 16:1* | up | FALSE | FALSE |
| LysoPC 16:0 | up | FALSE | FALSE |
| LysoPC 16:0(2n isomer) | up | FALSE | FALSE |
| LysoPE 20:4 | up | FALSE | FALSE |
| LysoPE 20:3 | up | FALSE | FALSE |
| LysoPC 17:2 | up | down | FALSE |
| LysoPE 20:2 | up | FALSE | FALSE |
| LysoPE 20:2(2n isomer) | up | down | FALSE |
| LysoPC 17:1 | up | FALSE | FALSE |
| 6'-O-Glucosylaucubin | down | FALSE | FALSE |
| LysoPC 17:0 | up | FALSE | FALSE |
| 1-α-Linolenoyl-glycerol-3-O-glucoside | up | up | FALSE |
| LysoPC 18:4 | up | FALSE | FALSE |
| 1,3-O-Dicaffeoylquinic Acid (Cynarin) | up | FALSE | FALSE |
| 1-Linoleoylglycerol-3-O-glucoside* | up | FALSE | FALSE |
| 2-Linoleoylglycerol-1-O-glucoside* | up | FALSE | FALSE |
| Bis(N,N-Diethylethanaminium)-2-acetamido-1,5-anhydro-2-deoxy-1-[-hydroxy(phosphonato)methyl]-D-glucitol | up | FALSE | FALSE |
| LysoPC 18:3(2n isomer) | up | FALSE | FALSE |
| LysoPC 18:3 | up | FALSE | FALSE |
| LysoPC 18:2(2n isomer) | up | FALSE | FALSE |
| Pinoresinol-4-O-glucoside | down | FALSE | up |
| LysoPC 18:1(2n isomer) | up | FALSE | FALSE |
| LysoPC 18:1 | up | FALSE | FALSE |
| LysoPC 18:0 | up | FALSE | FALSE |
| LysoPC 18:0(2n isomer) | up | FALSE | FALSE |
| 5,8,11,14-Pentadecanoamide | up | FALSE | FALSE |
| LysoPC 19:3 | up | FALSE | FALSE |
| LysoPC 19:2(2n isomer)* | up | down | FALSE |
| LysoPC 19:2* | up | down | FALSE |
| LysoPC 19:1 | up | down | FALSE |
| LysoPC 19:0 | up | down | FALSE |
| 2-Hydroxy-5,8,11,14,17-icosapentaenoyloxy]propyl-2-(trimethylammonio)ethyl phosphate | up | FALSE | FALSE |
| Propyl 2-(trimethylammonio)ethyl phosphate | up | FALSE | FALSE |
| LysoPC 20:3 | up | FALSE | FALSE |
| LysoPC 20:2 | up | FALSE | FALSE |
| LysoPC 20:1 | up | down | FALSE |
| LysoPC 20:0 | up | FALSE | FALSE |
| 5'-Methoxyisolariciresinol-9'-O-glucoside | up | FALSE | FALSE |
| Nuatigenin-3-O-glucoside | up | down | FALSE |
| Pennogenin-3-O-β-D-glucopyranoside | up | FALSE | down |
| Uridine 5'-diphospho-N-acetylglucosamine | up | FALSE | FALSE |
| 4-O-(6'-O-Glucosylcaffeoylglucosyl)-4-hydroxybenzyl alcohol | FALSE | up | FALSE |
| Chrysoeriol-5,7-di-O-glucoside* | up | FALSE | FALSE |
| Isorhamnetin-3,7-O-diglucoside | FALSE | up | FALSE |
| D(+)-Melezitose O-rhamnoside | up | FALSE | FALSE |
| O-Caffeoyl maltotriose | down | FALSE | FALSE |
| Nystose* | up | FALSE | FALSE |
| 3-Hydroxypropyl palmitate glc-glucosamine | up | FALSE | FALSE |
| 1-(9Z-Octadecenoyl)-2-(9-oxo-nonanoyl)-sn-glycero-3-phosphocholine | FALSE | up | FALSE |
| 2-α-Linolenoyl-glycerol-1,3-di-O-glucoside* | up | FALSE | FALSE |
| 1-Linolenoyl-rac-glycerol-diglucoside | up | FALSE | FALSE |
| 1-α-Linolenoyl-glycerol-2,3-di-O-glucoside* | up | FALSE | FALSE |
| 2-Linoleoylglycerol-1,3-di-O-glucoside* | up | FALSE | FALSE |
| 1-Linoleoylglycerol-2,3-di-O-glucoside* | up | FALSE | FALSE |
| 3-O-(2-O-Acetyl-glucosyl)oleanolic acid | up | FALSE | FALSE |
| Pinoresinol-4,4'-O-di-O-glucoside | FALSE | up | FALSE |
| 3,6'-Diferuloylsucrose | down | FALSE | FALSE |
| Diosgenin-3-O-α-L-rhamnosyl-(1→3)-β-glcoside | up | down | FALSE |
| Trillin-6'-O-glucoside | up | FALSE | FALSE |
| HydroxyDiosgenin-hamnosyl(1,2)glucoside* | FALSE | up | FALSE |
| HydroxyYamogenin-rhamnosyl(1,2)glucoside* | FALSE | up | FALSE |
| (25S)-Kingianoside A | up | FALSE | FALSE |
| Kaempferol-3-O-rutinoside-7-O-glucoside | FALSE | up | FALSE |
| Oleanolic acid-3-O-xylosyl(1→3)glucuronide | up | FALSE | FALSE |
| Kaempferol-3-O-sophorotrioside | FALSE | up | FALSE |
| Isorhamnetin-3-O-rutinoside-4'-O-glucoside | FALSE | up | FALSE |
| Isorhamnetin-3-O-(2''-O-glucosyl)galactoside-7-O-glucoside | FALSE | up | FALSE |
| Polyphyllin H | up | FALSE | FALSE |
| Pennogenin-3-O-xylosyl(1,3)Acetyl(1,2)rhamnosyl(1,2)fucosyl(1,2)glucoside | FALSE | up | FALSE |

**Table S6.** The quality of RNA-Seq of *P. polyphylla* seeds.

| Sample | Raw Reads | Clean Reads | Clean Base (G) | Error Rate (%) | Q20 (%) | Q30 (%) | GC Content (%) |
| --- | --- | --- | --- | --- | --- | --- | --- |
| S0-1 | 61128224 | 57835506 | 8.68 | 0.03 | 97.77 | 93.64 | 51.92 |
| S0-2 | 57877148 | 54259604 | 8.14 | 0.03 | 97.62 | 93.3 | 51.77 |
| S0-3 | 60029418 | 57406512 | 8.61 | 0.03 | 97.78 | 93.73 | 51.61 |
| S1-1 | 81008882 | 75797322 | 11.37 | 0.02 | 98.17 | 94.48 | 51.24 |
| S1-2 | 57673290 | 53956510 | 8.09 | 0.03 | 97.79 | 93.63 | 51.27 |
| S1-3 | 69033304 | 65642212 | 9.85 | 0.03 | 98.05 | 94.22 | 51.82 |
| S2-1 | 64798000 | 61360090 | 9.2 | 0.03 | 98.01 | 94.2 | 51.69 |
| S2-2 | 69129842 | 65325896 | 9.8 | 0.03 | 98.01 | 94.21 | 51.49 |
| S2-3 | 65419546 | 62554802 | 9.38 | 0.03 | 97.92 | 93.91 | 51.46 |
| S3-1 | 70480824 | 67380562 | 10.11 | 0.03 | 97.92 | 93.92 | 51.45 |
| S3-2 | 56572692 | 53835314 | 8.08 | 0.02 | 98.09 | 94.39 | 51.73 |
| S3-3 | 62581826 | 59181504 | 8.88 | 0.02 | 98.1 | 94.4 | 51.59 |

**Table S7.** Genes annotated in each public database

| database | number of genes | Percentage (%) |
| --- | --- | --- |
| KEGG | 14473 | 76.24 |
| NR | 17233 | 90.78 |
| SwissProt | 13546 | 71.35 |
| Trembl | 16922 | 89.14 |
| KOG | 11354 | 59.81 |
| GO | 14376 | 75.73 |
| Pfam | 15572 | 82.03 |
| Annotated in at least one Database | 17325 | 91.26 |
| Total Unigenes | 18984 | 100 |

**Table S8.** Different expressed genes (DEGs) in different pair-wise comparisons during the germination of *P. polyphylla* seeds.

| Group | down | up | false | total |
| --- | --- | --- | --- | --- |
| S0 vs S1 | 2445 | 6427 | 8405 | 17277 |
| S1 vs S2 | 3544 | 1150 | 12459 | 17153 |
| S2 vs S3 | 536 | 203 | 15104 | 15843 |
